# Using near–surface temperature data to vicariously calibrate high-resolution thermal infrared imagery and estimate physical surface properties

**DOI:** 10.1016/j.mex.2022.101644

**Published:** 2022-04-02

**Authors:** Timothy N. Titus, J. Judson Wynne, Murzy D. Jhabvala, Nathalie A. Cabrol

**Affiliations:** U.S. Geological Survey, Northern Arizona University, Goddard Space Flight Center, SETI Institute, United States

**Keywords:** Pisgah lava field, QWIP, Fourier transformation, Thermal conductivity, Planetary analog

## Abstract

Thermal response of the surface to solar insolation is a function of the topography and the thermal physical characteristics of the landscape, which include bulk density, heat capacity, thermal conductivity and surface albedo and emissivity. Thermal imaging is routinely used to constrain thermal physical properties by characterizing or modeling changes in the diurnal temperature profiles. Images need to be acquired throughout the diurnal cycle – typically this is done twice during a diurnal cycle, but we suggest multiple times. Comparison of images acquired over 24 hours requires that either the data be calibrated to surface temperature, or the response of the thermal camera is linear and stable over the image acquisition period. Depending on the type and age of the thermal instrument, imagery may be self-calibrated in radiance, corrected for atmospheric effects, and pixels converted to surface temperature. We used an experimental instrumentation where the calibration should be stable, but calibration coefficients are unknown. Cases may occur where one wishes to validate the camera's calibration. We present a method to validate and calibrate the instrument and characterize the thermal physical properties for areas of interest. Finally, in situ high-temporal-resolution oblique thermal imaging can be invaluable in preparation for conducting overflight missions. We present the following:

• The use of oblique thermal high temporal resolution thermal imaging over diurnal or multiday periods for the characterization of landscapes has not been widespread but poses great potential.
• A method of collecting and analyzing thermal data that can be used to either determine or validate thermal camera calibration coefficients.
• An approach to characterize thermophysical properties of the landscape using oblique temporally high-resolution thermal imaging, combined with in situ ground measurements.

## Graphical abstract

**Figure:**
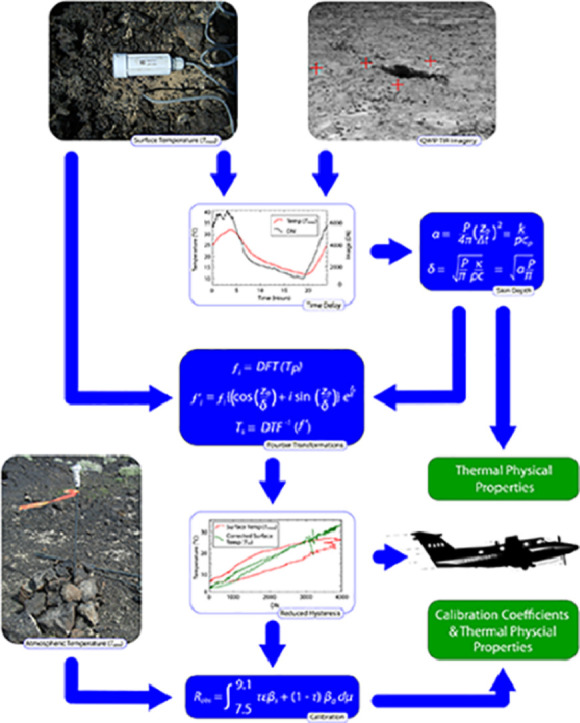

Specifications table

**Table:** 

Subject Area:	Earth and Planetary Sciences
More specific subject area:	*Thermal camera calibration, thermal physical surface properties*
Method name:	Using near–surface temperature data to vicariously calibrate high-resolution thermal infrared imagery and estimate physical surface properties
Name and reference of original method:	Infrared camera calibration; H. Budzier and G. Gerlach (2015) Calibration of uncooled thermal infrared cameras, J. Sens. Sens. Syst., 4, 187–197, 2015 doi:10.5194/jsss-4-187-2015
Resource availability:	(1) *The data used*https://doi.org/10.5066/P9PN5BMK*; (2) Supplementary online material*

## *Method details

### Background

Remote sensing has been used to characterize landscapes (e.g., the identification of possible energy or mineral deposits, the classification of land use) and to form a baseline for determining future change detection (e.g., effects of climate change or urban expansion). On other planets, remote sensing is the primary tool for understanding geological processes and landscape evolution. Typically, remote sensing is conducted from orbital platforms where viewing angles are only a few degrees from surface normal. However, remote sensing can also be conducted from stationary landers, rovers, and drones, where oblique views become the norm. Wavelength ranges used for passive remote sensing include visible and near-to thermal infrared. For oblique imagery using reflected light, knowledge of the surface photometric function is an important component for analysis. For imagery using thermal emission, it is usually assumed that the thermal emission is isotropic. For oblique views, this assumption may not valid. The focus here is the use of thermal infrared oblique observations of cave-bearing volcanic landscapes.

The use of thermal cameras to analyze landscapes has expanded over the last decade (e.g., [6,14,15,21,[32], [33], [34],^39^,^40^]). However, depending on the type and age of the thermal cameras, the output image may be self-calibrated in radiance, corrected for atmospheric absorption and emission, and each pixel converted to surface temperature values. However, we used an experimental thermal camera where the calibration is reported to be stable with a linear response, but calibration coefficients were either unknown or poorly constrained. Proper calibration of thermal infrared cameras should be conducted under laboratory conditions but is also a time-consuming complicated process (e.g., [4]). The approach presented here is a simplified method of vicarious in situ calibration, based on field data collected using an experimental Quantum Well Infrared Photodetector (QWIP) thermal camera.

In 2010, we conducted a series of experiments using the QWIP thermal instrument, which was an experimental precursor version of a QWIP thermal camera ([17,19] that has been flown on the International Space Station and is the thermal imaging instrument onboard Landsat 8 [18,20] and Landsat 9 [25]. Several experiments to analyze cave-bearing landscapes in the Mojave Desert, California, assisted in this instrument's maturation. Wynne et al., [40,42] reported thermal distinctions between cave, tunnel cave (i.e., a subterranean feature with entrances on either end, typically with frequent air flow) and random non-cave locations on the surface in thermal images captured at 10‐minute intervals over a 24‐hour period. Their results demonstrated how larger caves may be distinguishable from shallow alcoves and tunnel features. Using a similar dataset of thermal imagery, Titus et al., [34] further examined multiple thermal images containing cave entrances; they found the detectability of caves was best when multiple thermal images were acquired either at the hottest (early afternoon) or coolest (pre-dawn) times of day. Moreover, they reported that combining multiple images captured over a 24-hour period yielded the best results.

The methods presented here were prompted by a QWIP-based airborne thermal imagery acquisition mission conducted in 2011 (refer to [41,42] for details). As the acquired imagery were not radiometrically calibrated and atmospherically corrected, we needed to confirm the Digital Number (DN) pixel values were valid as input into algorithms for detecting terrestrial caves. Fortunately, we had previously collected a 2010 dataset of QWIP ground-based thermal imagery and in situ near surface kinetic temperature data [34,35], as a precursor experiment to the overflight campaign [16,42]. We used these data to vicariously calibrate thermal infrared imagery, as well as estimate physical surface properties. As these data were simultaneously acquired at a temporally high resolution over a diurnal cycle, we developed and applied the techniques presented here to both extract in situ thermal physical properties and calculate camera calibration coefficients.

### Study Area

Our experiment was conducted at Pisgah lava field, which is located about 175 miles northeast of Los Angeles on Bureau of Land Management lands. Consisting of Quaternary basaltic lava and a cinder cone superimposed on alluvial deposits and lacustrine sediments of Lavic Lake playa [8], the flow is approximately 21,000 years old [38]. Extending ∼18 km to the west and 8 km to the southeast from the Pisgah cinder cone [13], three eruption phases emitted both pāhoehoe and aʻa lava, which vary in thickness from 1 to 5 meters across the field [9]. In addition to the lava field, sections of the landscape were punctuated by desert pavements and sand deposits, which made the area ideal for thermal imaging across a range of thermal physical properties. We used the cinder cone as our vantage point for acquiring oblique imagery.

**Fig. 1:**
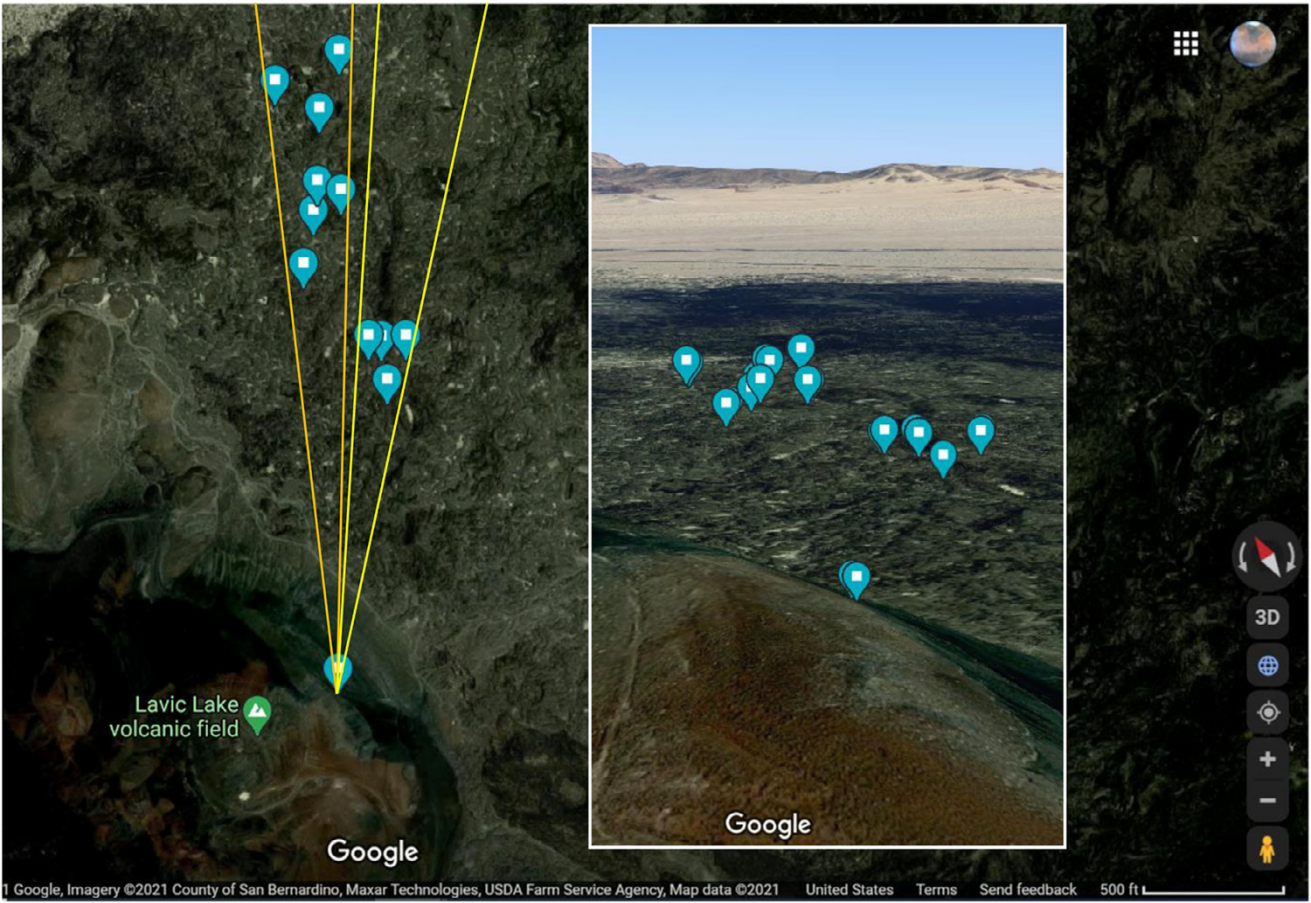
Pisgah lava field, Mojave Desert, California, with QWIP camera location and two regions of interest identified. Orange vectors approximate the left and right field of view for B cave dataset, while yellow vectors approximate the left and right limits of the field of view for Station 7 trench dataset. Blue balloons denote camera and sensor locations. The inset shows the same scene but at an oblique angle and provides additional context. Credit: Google Earth.

**Fig. 2:**
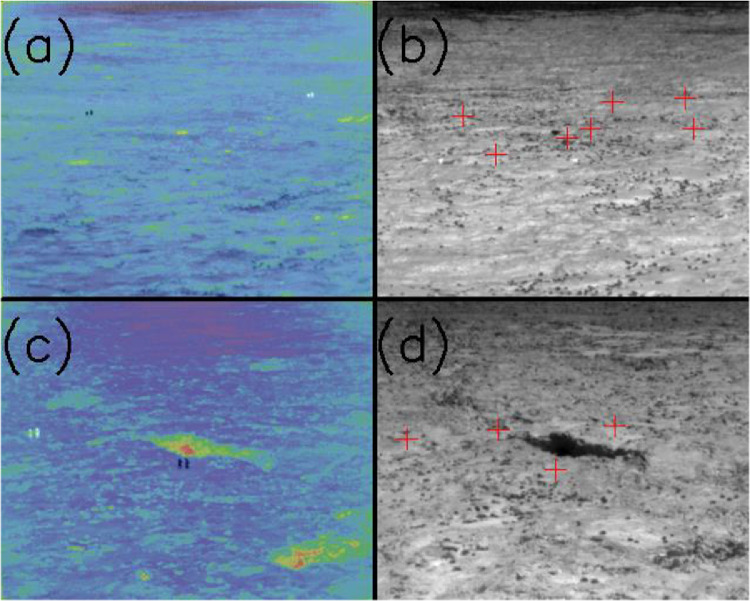
(a) Differenced images show location of two sensor locations in the B Cave ROI. (b) Sensor locations for all seven sensors surrounding B Cave. (c) Differenced images show location of two sensor locations in the Station 7 trench ROI. (d) Sensor locations for all four sensors surrounding Station 7 trench.

Fig. 1 depicts the study area with camera location and the two regions of interest (ROI). Fig. 2 shows sensor locations within the camera's field of view. Thermal imaging of each ROI was conducted on consecutive days. We collected imagery of the B Cave ROI first and Station 7 trench ROI was acquired the next day. Tables 1 and 2 describe the metadata associated with the camera and the in situ sensors, which include GPS coordinates.

**Table 1:** Regions of interest and time periods of simultaneous data acquisition (GMT -7).

ROI	Start Date	Start Time	End Date	End Time	No. Images
B Cave	2010-03-23	10:15:04	2010-3-24	10:10:06	288
Station 7 trench	2010-03-24	12:00:06	2010-3-25	11:55:06	287*

⁎ Note: Station 7 trench dataset is missing the image that was to be acquired at 3:05:06. Therefore, the interval between *180.tif and 181.tif is 10 min for Station 7 trench.*

**Table 2:** Sensor and camera locations. ID is either the QWIP camera or the sensor serial number. Locations of sensors were determined using a Garmin Etrex (Vista HCx) GPS and WGS 84 datum. Time averaging was used to increase accuracy to ∼3m horizontal. Vertical errors are typically 5x the horizonal error for this type of GPS, suggesting the elevation error is ∼15m. Pixel sample and line number represent coordinates of the sensor within the QWIP image. The estimated uncertainty in distance is ∼5 m, while the estimated uncertainty in angle is ∼30%.

ID	Longitude	Latitude	Elevation (m)	Pixel Sample	Pixel Line	Distance (m)	Elev. Angle
QWIP Camera	W116.37409	N34.74651	764	n/a	n/a	n/a	n/a
Atmospheric Temperature/Humidity							
9702167	W116.37125	N34.75059	701	184	146	526.498	6.840
9702171	W116.37182	N34.74858	710	154	111	314.623	9.786
B Cave (23-24 March 2010)							
9695783	W116.37192	N34.74998	707	103	124	437.560	7.443
9695779	W116.37148	N34.75037	699	164	138	494.978	7.502
9695787	W116.37125	N34.75059	701	184	146	526.498	6.840
9695785	W116.37107	N34.75039	706	272	146	515.076	6.438
9695788	W116.37077	N34.75118	707	203	169	603.545	5.403
9695782	W116.37103	N34.75166	693	75	157	640.735	6.336
9695786	W116.37019	N34.75157	697	265	172	669.034	5.728
Station 7 trench (24-25 March 2010)							
9695781	W116.37182	N34.74858	710	154	111	314.623	9.786
2233224	W116.37173	N34.74904	709	27	137	358.665	8.752
2233225	W116.37135	N34.74885	709	205	148	365.109	8.598
2041160	W116.37160	N34.74897	707	105	145	360.402	9.024

Data collected from B Cave was acquired at a high oblique angle with the center of the image being ∼ 7.5°, measured from the horizontal. Sensors within the image ranged from 7.5° to 5.4° (near the image background). Distance between camera and sensors varied from ∼440m to 670m, which would also suggest a range in air mass values (the amount of air along the line of sight) were present. Importantly, the distance of 670 m was not far from the expected altitude for possible overflights.

Data collected from Station 7 trench were acquired the day after B Cave data were collected (Table 1). However, these data were collected at a less oblique range of angles of elevation, with the center of the image being ∼ 9°. Sensors located within the image (Table 2) ranged from 8.6° to 9.8°. Distance between camera and sensors varied from ∼315m to 365m. Distances from the camera to the four sensors were similar; thus, the air mass was also similar.

### QWIP thermal instrument specifications

The QWIP thermal infrared sensor responded to thermal infrared radiation from 7.5 to 9.1 µm with a peak response of 8.7 µm. The detector array was cooled with a miniature Stirling cycle cryocooler to 67K (-206°C), which minimized both detector noise and internal field of view optical effects. The QWIP used a standard 50mm Infrared (IR) lens and had an instantaneous field of view of 8.8° x 11°. Integration time was 0.0164 sec. The camera was preset to remotely capture one 16-bit image every 5 minutes for a 24-hour period (or 288 images total). Each image is in a 320 × 256 pixel format where each detector pixel within the array is a 30 µm square. Although the imagery was not radiometrically calibrated, the instrument had the ability to resolve signals from objects where temperature variations are less than 0.02°C. Calibration tests on QWIP thermal infrared cameras have been documented to be stable for months, even years [20].

### Ground-based temperature instrumentation

We used ONSET Hobo logger U23-003 to measure surface kinetic temperatures. Accuracy was ±0.21°C from 0° to 50°C with a resolution of ∼0.02°C. Sensor response time when located in soil and rock was not provided but was reported to be 30 seconds when located in stirred water or three minutes in air moving at 1 m/sec (https://www.onsetcomp.com/products/data-loggers/u23-003/). Temperature sensors used to measure the atmosphere were ONSET Hobo logger U23-001. The accuracy of the U23-001A is ±0.2°C from 0 to 70°C, with a resolution of ∼0.04°C. Response time was reported to be 10 minutes in air moving at 1 m/sec. The accuracy of the U23-001A for relative humidity (RH) is typically ±2.5% between 10% and 90% RH, with a maximum of ±3.5% including hysteresis at 25°C. At HR below 10% and above 90%, the accuracy is typically ±5%. (https://www.onsetcomp.com/products/data-loggers/u23-001/)

### Experimental design

Proper experimental design is key to success. This section describes our methodology, but we also provide both recommendations and sampling improvements for guiding similar studies. We believe that possible applications of high temporal resolution diurnal oblique thermal imaging include landscape characterization on other planets (e.g., Mars) using a rover-mounted camera, collection of data that can be used for overflight planning (such as the original purpose of this study), and thermal physical characterization of landscapes where overflights, even by drones, are either not practical or not allowed.

When collecting long term thermal infrared imagery (i.e., over a 24-hour period) in backcountry settings, the following considerations should be applied. The camera should be mounted in a stable location and in a secure manner to ensure all imagery in the sequence can be co-registered. As imagery is acquired, the camera's field of view (FoV) should not shift by more than a fraction of a pixel to avoid having to register the images. Thus, the mounting apparatus must be stable and data acquisition during gusty wind events should be avoided. Importantly, care should be taken when personnel are moving around the instrument to avoid any accidental jostling – as this could compromise data collection and quality. When capturing data over a diurnal period, we recommend a frame acquisition rate of every 5 to 10 minutes. Our experiment showed a rate of every five minutes was temporally sufficient ([34,35]; this study). Higher rates of image acquisition could be used to test this assertion and/or for better characterization of the instrument properties, such as signal-to-noise ratio (SNR) or Noise Equivalent Delta Temperature (NEDT).

Instrumentation for ground measurements should be deployed in areas within the camera's FoV. Sensors should minimally sample temperature at the same frame acquisition rate, but ideally at least every minute. For our study, temperature probes were inserted into the soil (or rock) so that the base of the sensor was flush with the surface. When installing into rock, we used a hammer drill with a bit the same diameter as the temperature probe (5mm). Temperature data acquired effectively represented the near surface temperature at the midpoint of the probe. By inserting the probe into the soil or rock, we minimized the effect of direct insolation on the probe. However, this introduced a time lag and a small amount of attenuation between the actual surface temperature (as captured by the camera) and the ground temperature measured by the probe. However, this time delay can be used to estimate both the surface thermal physical properties (e.g., thermal diffusivity) and the attenuation of actual surface temperature. Additionally, the time delay and the calculated attenuation can be used to correct the measured ground temperature to the actual surface temperature. Thereafter, this corrected surface temperature can be directly compared to a thermal camera's digital number (DN) output for the pixels containing ground instrument locations.

Ground temperature sensors should be placed in areas uniform in slope, composition, and texture, and correspond to several neighboring pixels within the camera's FoV. This will help reduce possible errors with sensor location within the imagery. For this study, we also restricted the slopes to within a few degrees of zero (i.e., flat or horizontal surfaces). Refer to figures S1 through S11 for examples of terrain types where sensors were installed.

To directly compare measured ground temperatures to thermal DN levels, ground sensor locations within the thermal images were needed, i.e., image line and sample. We used the observed heat from our own bodies in images to mark locations of sensors in a few of the pre-dawn images. When these images were acquired, field personnel straddled the sensor, with the instrument between their feet. This was repeated for all instruments and was quite effective. The contrast between the “warm bodies” and the surrounding area can be enhanced by taking the difference between the image of interest and either the previous or the next image in the sequence. Fig. 2 is an example of this method where two sensor locations can be identified.

While not needed for the near surface temperature corrections, atmospheric temperature and humidity was required for our in situ calibration technique to be most effective. Therefore, we collocated the temperature/humidity (U23-001) sensors proximal to the in situ near surface sensors. These additional sensors were positioned approximately one meter above ground (see Figure S3 for example image). A makeshift sunshade, constructed with aluminum foil, was used in an attempt to minimize the effect from direct sunlight and surface radiance on the temperature probe. In retrospect, we should have sampled atmospheric temperature at a height of 1.5 to 2 meters above the ground surface and used commercially available sunshades. This would have optimally minimized the effects from both direct sunlight and surface emission and reduced the presumably large temperature effects of the dark-colored and thermally conductive iron rebar – used as the mounting apparatus for the surface temperature probes.

Another methodological refinement would have been to place two instruments at different heights to ascertain the appropriate height for these sorts of experiments. While tracking the atmospheric temperature at one location per ROI was valuable, additional locations (e.g., foreground and background) would have allowed for a more complete characterization of the different air masses.

### Thermal diffusion theory

Near surface temperature, as a function of depth, can be determined by solving the thermal diffusion equation:

(1) $$\frac{dT}{dt}=\;\alpha \;\frac{\partial^{2}T}{\partial z^{2}}$$

where *α=k/ρc*, α is the thermal diffusivity, *k* is thermal conductivity, *ρc* is the density times the heat capacity, *T* is temperature and *z* is depth into the subsurface.

**Fig. 3:**
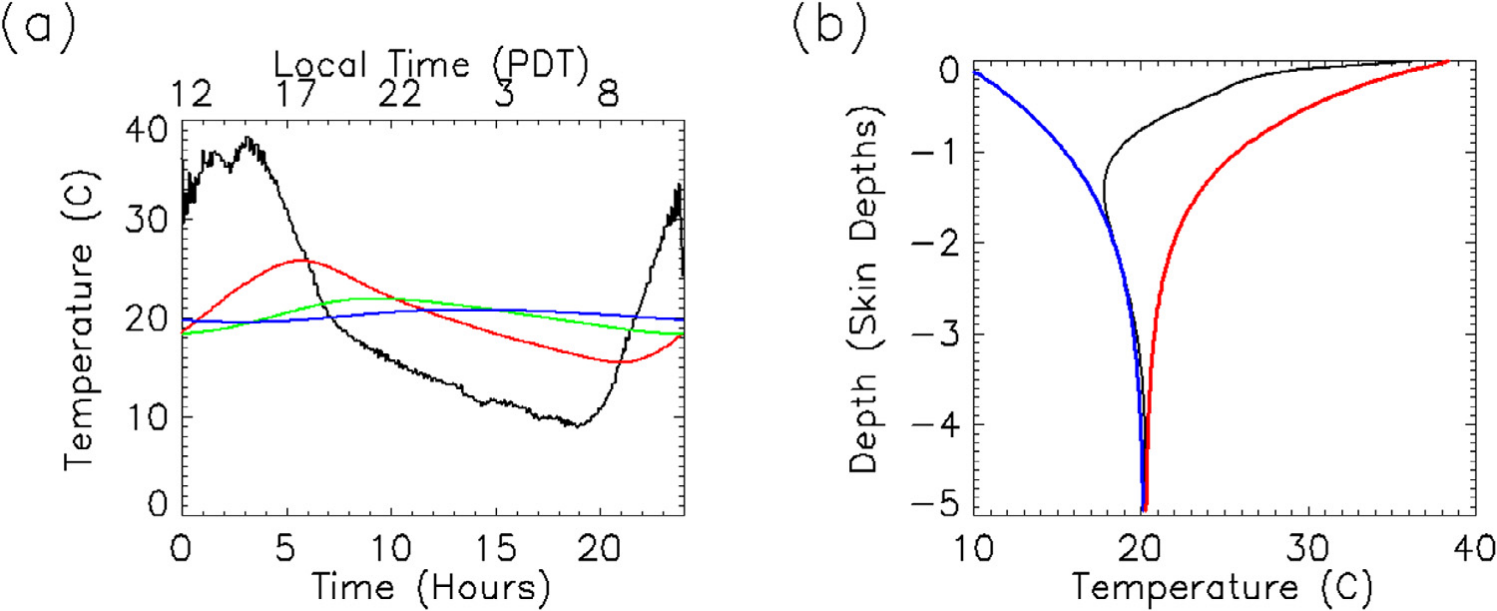
Thermal profile examples: (a) Temperature vs. time at several depths, z. The black line represents the surface. Red, green, and blue lines show temperature at one, two, and three diurnal skin depths, respectively. (b) Diurnal minimum and maximum temperatures shown as a function of depth. Black curve is the temperature profile at noon (local daylight time). Blue and red dashed lines represent the minimum and maximum temperature envelopes, respectively.

Since surface temperature is cyclic, one convenient and useful solution to this 2^nd^ order PDE is:

(2) $$T\left(t,z\right)=T_{o}+\sum_{i}e^{-z/\delta_{i}}\;A_{i}cos\left(\theta_{i}\left(t,z\right)\;+\varphi_{i}\right)$$

where *θ_i_*(*t,z*) *=* (2*πi/P*)*t - z/δ_i_* and *φ_i_* is the initial phase shift of the surface temperature, *t* is time, *T_o_* is the diurnal mean surface temperature, and *A_i_* is the amplitude of the temperature variation of the *i*^th^ harmonic. Thermal skin depth, *δ_i_*, is a measure of how far a surface temperature cycle of an arbitrary period penetrates the regolith. The exact definition is the depth at which the amplitude of the thermal wave (over a given period) is attenuated by a factor 1/*e*.

(3) $$\delta =\;\sqrt{\frac{P}{\pi }\;\frac{k}{\rho c}}=\;\sqrt{\alpha \;\frac{P}{\pi }}$$

where *δ* is skin depth, and *P* is the period of the cycle (86,400 sec for the terrestrial diurnal cycle). An example of this is shown in Fig. 3.

### Thermal diffusion analysis

**Fig. 4:**
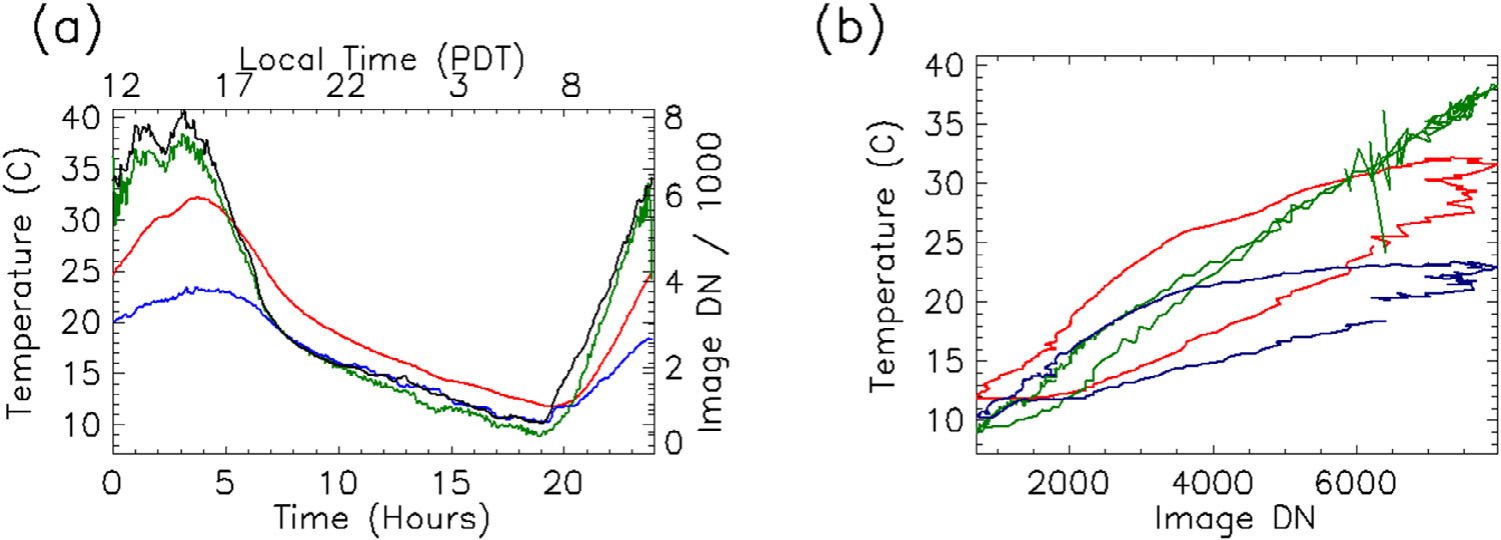
Sensor 9695781 thermal profile of raw data before and after the Fourier transform (FT) correction. (a) Temperature vs. time with curves shown as measured rock temperature (red), the FT corrected temperature (green), and atmospheric temperature (blue). Black line represents the thermal image pixel DN values. (b) Temperature vs DN, with the red line representing the uncorrected temperature vs. thermal image pixel DN values. The green line represents the FT corrected temperature values vs. thermal image pixel DN values. The green line demonstrates hysteresis between measured temperature and observed radiance. The green line is better represented by a best-fit line, as much of the hysteresis has been removed. Some hysteresis remained at the lower DN values, which could be a result of atmospheric radiance as shown in the blue line.

Once a diurnal cycle of data has been acquired, pixels corresponding to in situ sensor locations should be identified. By plotting each identified pixel's DN levels and in situ near surface temperature versus time (see Fig. 4), a quick estimate of the time lag between image signals and measured temperatures can be calculated. Once the time lag has been determined, the following equation can be used to derive the thermal diffusivity,

(4) $$\alpha =\frac{P}{4\pi }\;{\left(\frac{z_{0}}{\Delta t}\right)}^{2}=\;\frac{k}{\rho c}$$

where *P* is the number of seconds in a diurnal cycle, Δt is the time delay between the image acquisition and the corresponding ground instrument temperature, and *z*_o_ is the depth to the midpoint of the temperature probe.

A few assumptions should be considered with this calculation. First, a possible source of error may occur between the thermal contact between the probe and the surrounding material. Poor thermal contact will increase the observed time lag, and therefore decrease the estimated thermal diffusivity. Sensor lag is another potential source of error, ∼30 seconds based on the sensor specifications. Therefore, our estimates may represent the lower limits of these values and have the most effect for smaller time lags (higher thermal diffusivity).

Robertson [31] provided a review of thermal physical properties for rock including those relevant to lava fields. Typically, basalt has an α of 9 × 10^−7^ m^2^/s. Our highest estimated coefficient of diffusion was 18 × 10^−7^ m^2^/s, which was closer to granite than to pāhoehoe. It is unlikely that an error in the time delay estimate could compensate for a factor of two increase in measured diffusivity when compared to laboratory measurements. Perhaps the deployed in situ sensor estimated temperature is not representative of the radiance as observed by the QWIP at this location. Because the U23-003 sensors are equipped with two external probes, future deployments should acquire two in situ temperatures instead of just one.

While α, the thermal diffusivity, is the physical property that we directly estimate from the Δt, the time delay between the imaging and the sensor probes’ reaction to changing insolation, thermal inertia (Γ) is the thermal physical property most used to characterize landscapes [37]

(5) k=αρc

(6) $$\Gamma =\sqrt{k\rho c}=\;\sqrt{\alpha }\rho c$$

where *ρ* is density and *c* is the heat capacity for basalt. The product (*ρc*) of density (*ρ*) and heat capacity (*c*) typically only varies by a factor of two or less for most surface materials [22,27,30,36]. However, thermal conductivity can vary by several orders of magnitude. If one either knows or can reasonably estimate the surface material density and heat capacity, then both thermal conductivity (*k,* Eq. 5) and thermal inertia (Γ, Eq. 6) can be determined. We used these relationships, combined with Eq. 4, to estimate thermal conductivity and inertia values presented Table 3. We assumed a density and heat capacity consistent with basalt [31]. Estimated values were derived from the time-delay shown in Table 3. Unfortunately, we were not able to ground truth these properties.

**Table 3:** Derived thermal physical properties at sensor locations. ID is the sensor number. Time delay represents the temporal shift applied between the DN profile and the sensor temperature profile. α is the coefficient of diffusion, while k is the derived thermal conductivity (assuming that ρc volumetric heat capacity is 2.08 × 10^−6^ J m^−3^ K^−1^). Γ is the estimated thermal inertia in standard SI units. Figure number (#) references supplementary online material that includes images of each sensor site. An asterisk indicates this was also the location where air temperature and humidity data were collected.

Sensor ID Number	Time delay (mins)	α (x 10^−7^ m^2^/s)	Normalized Diffusivity α/α_max_	k	Γ	Figure #
B Cave ROI						
9695783	36	3.71384	0.197531	0.172102	282.407	S1
9695779	75	0.85567	0.0455111	0.00913591	31.2319	S2
9695787	22	9.94451	0.528926	1.23397	1237.41	S3*
9695785	41	2.86326	0.15229	0.102297	191.175	S4
9695788	46	2.27464	0.120983	0.0645603	135.366	S5
9695782	71	0.954799	0.0507836	0.0113753	36.8135	S6
9695786	34	4.16362	0.221453	0.216312	335.232	S7
Station 7 trench ROI						
9695781	77	0.811797	0.0431776	0.00822307	28.8609	S8*
2233224	47	2.17888	0.11589	0.0592387	126.908	S9
2233225	16	18.8013	1	4.41079	3216.79	S10
2041160	19	13.3328	0.709141	2.21811	1920.97	S11

### Thermal diffusion correction for surface temperature

While the thermal diffusivity (*α*) is a constant, skin depth is a function of the period over which change occurs. This means the temperature attenuation correction must be conducted over a range of time periods. The most direct approach is to use a discrete Fourier transform (DFT) to identify amplitudes and phase shifts (which can be represented as a complex number) for the entire range of periods represented in the temperature time sequence. These amplitudes and phase shifts should be corrected using Eq. 7.

(7) $$\begin{array}{c} f_{i}=DFT\left(T_{p}\right)\\ {f^{\prime }}_{i}=f_{i}\;\left\{(cos\;\left(\frac{z_{0}}{\delta_{i}}\right)\;+j\;sin\;\left(\frac{z_{0}}{\delta_{i}}\;\right)\;\right\}e^{\frac{z_{0}}{\delta_{i}}}\\ T_{s}=DFT^{-1}\left(f^{\prime }\right) \end{array}$$

Where *T_p_* is the temperature measured by the sensor probe, *T_s_* is the corrected surface temperature, and an inverse Fourier transform (DFT^−1^) can then be used to convert the corrected spectrum back to the temperature time sequence. d_i_ is the harmonic specific skin depth and is defined by Eq. 3, except the period, *P*, is replaced by *P*/*i*. Fig. 4 illustrates this technique applied to field data. This approach also amplifies higher frequency noise contained in the data, so this technique should only be applied if probe depth is less than the diurnal skin depth.

A comparison of measured surface temperatures and corrected surface temperatures compared the observed QWIP DNs are shown in Figs. 4, S1-S11. A hysteresis effect is clear in the uncorrected temperature data, but some residual hysteresis remains even in the corrected surface data. One possible cause for this residual hysteresis could be that the corrected surface temperature may not be completely representative of the entire surface area within a pixel's FOV. For example, scattered rocks could alter observed radiance by casting shadows or having different temperatures than the surrounding flat surface. Another possible cause is that the QWIP DN observations have not been corrected for atmospheric effects in these plots. The atmosphere is warmer during the morning and cooler during the night for the same corresponding surface temperatures, causing the observed DN levels to be higher in the morning and lower during the night.

### Atmospheric absorption

Even in an arid desert environment, the atmosphere contains water vapor that absorbs and re-emits thermal radiation. The amount of absorption and emission is a function of temperature, relative humidity, and distance between the thermal source and the detector. Using the formula in Minkina and Kleccha [24] and water vapor opacity tables from [10,28] (as used by Minkina and Kleccha [24]), we estimated atmospheric transmissivity (τ) as a function of air temperature, relative humidity, and distance using the standard exponential decay function

(8) $$\tau =\;e^{-h/h_{o}}$$

**Fig. 5:**
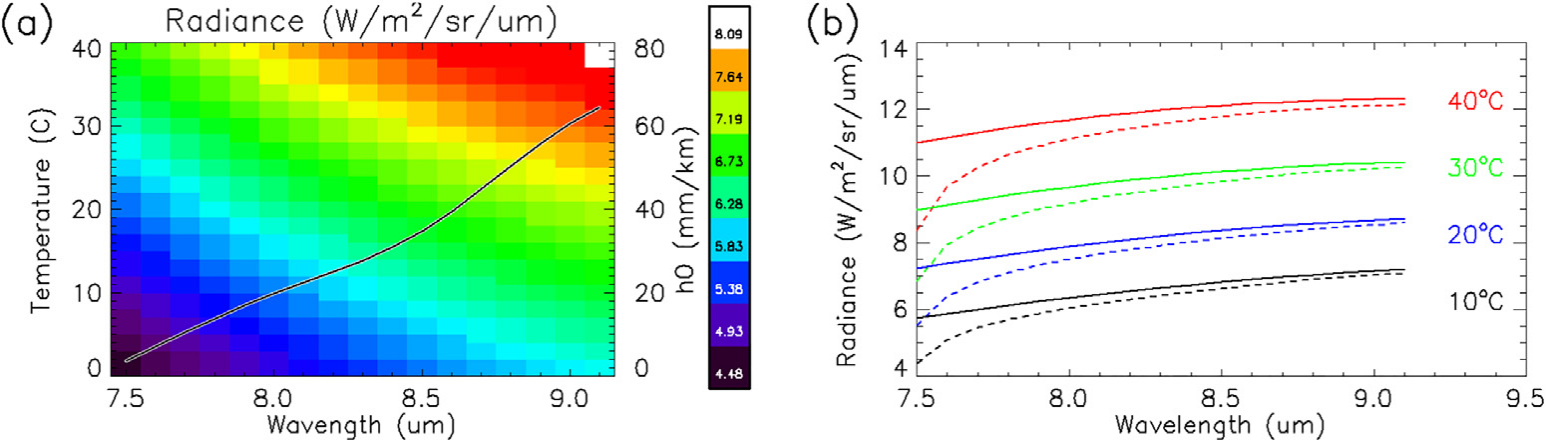
QWIP spectral window, Planck functions, and atmospheric absorption. (a) Blackbody radiance for the QWIP's spectral window as a function of temperature. The black line is h_0_, the characteristic water column used to estimate atmospheric transparency. (b) Radiance vs. wavelength as a function of temperature. Solid lines are the radiance levels for a blackbody and the dashed lines are the attenuated radiance of the blackbody radiance if looking through a 1mm/km equivalent water column. For reference, the range of an equivalent water column, h, for this data set was between 0.5 and 2 mm/km.

The coefficient, *h_o_*, is weighted assuming a top hat spectral response for the QWIP (8.5 – 9.1 µm) and are shown in Table 4a. Absorption due to atmospheric CO_2_ is negligible at these wavelengths [10,28]. Fig. 5 contains absorption coefficients and the Planck function for a range of temperatures and humidity within the QWIP spectral window. *h* is a function of temperature, relative humidity, and distance.

(9) $$h=\left(c_{0}+c_{1}T+c_{2}T^{2}+c_{3}T^{3}\right)rd$$

where *h* is the water column in mm/km, *T* is temperature (in degrees Celsius), *r* is relative humidity (from 0 to 1), and *d* is the distance in km. Coefficients *c_0_* thru *c_3_* are presented in Table 4b.

**Table 4a:** Atmospheric characteristic water column equivalent, h_0_.

Wavelength (um)	7.5	8	8.5	9	9.5
h_o_ (mm/km)	3.67950	19.8340	34.7064	60.5592	73.8088

**Table 4b:** Atmospheric coefficients to calculate water column equivalent, h.

Coefficient	c_0_	c_1_	c_2_	c_3_
Value	5	3.8333 × 10^−1^	10^−2^	1.6667 × 10^−4^

As previously mentioned, atmospheric temperature data were acquired one meter above the surface. In retrospect, a minimum of two meters may have improved our results. As it currently stands, a comparison of the Fourier transforms’ phase shifts of atmospheric and surface data suggested that “atmospheric” temperatures represented a significant component of surface radiance. We posit that acquiring temperature data at a height at or above two meters and the use of commercially available sunshades would have reduced this effect. Additional air temperature sensors along the line of sight should also be considered. The largest uncertainty in our calibration is due to an incomplete characterization of the line-of-sight atmosphere.

### Camera calibration

Once near surface temperatures have been corrected for both time delay and attenuation effects, these values can be converted to radiance using the Planck function – assuming the top hat spectral response function (i.e., uniform response across the spectral band-pass) ranges between 7.5 and 9.1 µm. These values can be compared directly to the corresponding DNs within the imagery. Fig. 4b illustrates a direct comparison where lower temperatures (corresponding to nighttime imagery) and higher temperatures (corresponding to daytime imagery) are depicted as lines with different slopes. This effect is likely due to atmospheric absorption (and lower thermal re-emission) along the line of sight. Assuming the detector array is linear and uniform in response, we used Eqs. 10 and 11 to determine both the camera's calibration coefficients and the surface emissivity at each sensor location.

(10) $$N=N_{1}R_{obs}+N_{0}$$

where *N* is the image DN, *N*_1_ is the calibration coefficient (gain) in DN per W•sr^−1^•m^−3^ (described below), *N*_0_ is the corresponding calibration offset, and *R_obs_* is the observed radiance. These equations did not account for the effects from the camera optics, but because the QWIP is cooled and used a standard IR lens, we assumed these effects were minimal.

(11) $$R_{obs}=\int_{7.5}^{9.1}\tau \varepsilon \beta_{s}+\left(1-\tau \right)\beta_{a}\;d\mu$$

where τ is the atmospheric transparency along the line of sight between the camera (Eq. 8) and the surface, *ε* is the surface emissivity, β*_s_* is the Planck function radiance as determined from the corrected surface temperature (Eq. 7), and β*_a_* is the atmospheric radiance. All terms are functions of wavelength and must be integrated across the QWIP spectral response function, which we assume is a top hat for this analysis.

Eqs. 10 and 11 can be combined:

(12) $$N=N_{1}\int_{7.5}^{9.1}\tau \varepsilon \beta_{s}+\left(1-\tau \right)\beta_{a}\;d\mu +N_{0}$$

This Eq. must be solved where the calibration coefficients, *N*_0_ and *N*_1_, are constant for all sites, and ε is a function of each site and should be restricted to physically reasonable values. In retrospect, using a TIR field spectrometer on site or returning samples to the lab for characterization to determine surface emissivity at oblique viewing angles would have further improved our calculations.

The use of multivariate linear regression techniques proved to be unwieldy, so Amoeba (an IDL minimization function based on the downhill simplex method [26,29]) was used to determine *N*_0_ and *N*_1_, as well as individual emissivity values for each of the 11 sites. We applied this process on the complete dataset. We also ran this procedure individually for both the B Cave and Station 7 trench ROIs.

### Method validation

The best fit calibration coefficients using the entire dataset were -9,304.05 DN and 1,838.57 DN/radiance unit for *N*_0_ and *N*_1_, respectively. Derived surface emissivity for the 11 sites, which were also free parameters where the solution was constrained to be between 0.55 and 1.0, ranged between 0.69 and 0.78, which were lower than previous laboratory measures of basaltic emissivity when observed at oblique angles (e.g., ε ∼ 0.86 at 10° viewing angle, [2]). Unfortunately, we were not able to ground truth these properties. For future experiments, we recommend using a field thermal infrared (TIR) spectrometer or returning samples to laboratory facilities to measure surface emissivity, especially at oblique angles. In addition, because emissivity was the only free parameter specific to each of the 11 sites, it is possible that any systematic lateral variations in atmospheric conditions or non-uniformity of the surface (e.g., scattered rocks) could be compensated for by altering the apparent effective emissivity. This is further discussed in the next section.

**Fig. 6:**
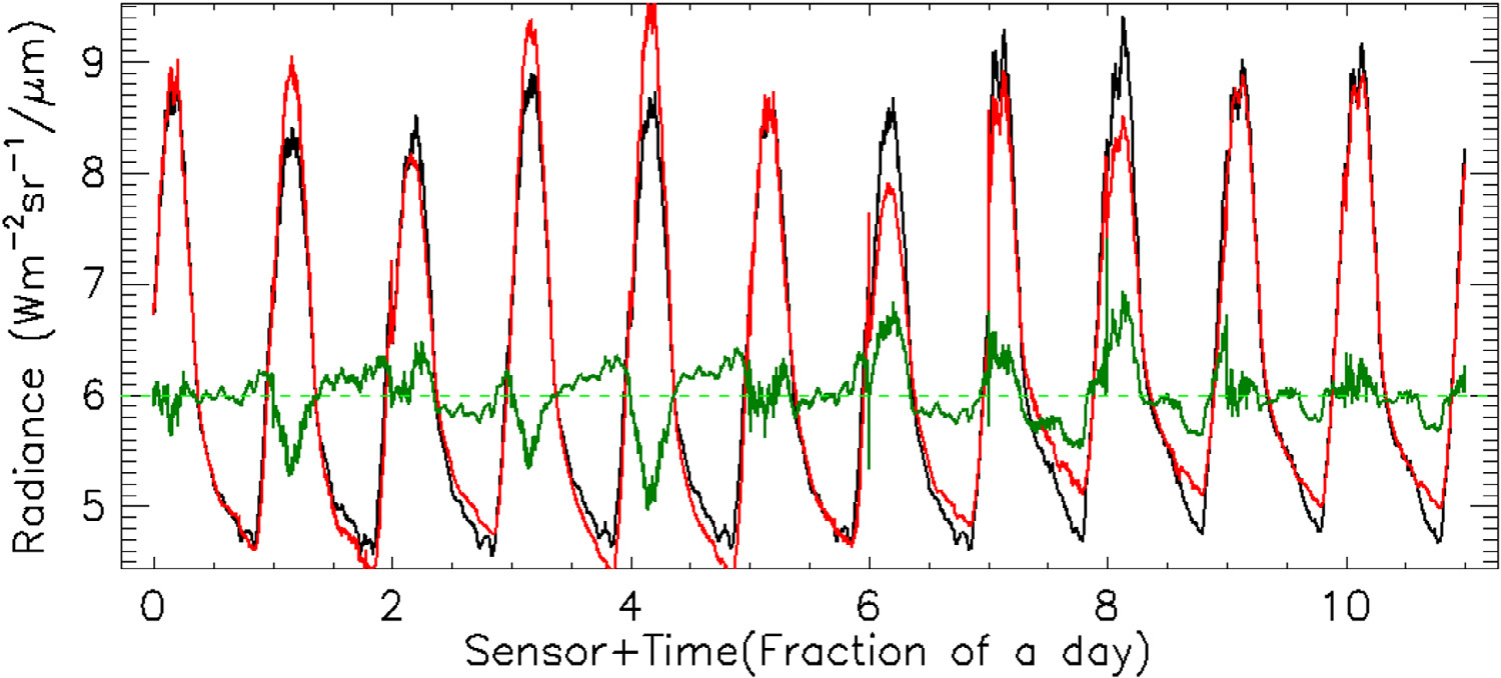
Calibration model compared to observations. The black line is the estimated observed radiance derived from the DN profiles at the 11 pixels (sensor sites), using the N_0_ (DN offset) and N_1_ (DN gain). The red line is the estimated radiance as derived from the corrected surface temperature, estimated emissivity, atmospheric absorption, and emission. The green line is the difference between these two estimates. Zero line has been offset to 6 Wm^−2^sr^−1^µm^−1^.

Calibration coefficients for the QWIP as determined for B Cave ROI had values of -8,727.04 DN and 1,864.02 DN/radiance unit. This was a change of 6.2% and 1.4% for *N*_0_ and *N*_1_, respectively. Calibration coefficients for the QWIP as determined for the Station 7 trench ROI had the values of -10,521.1 DN, 2,077.23 DN/radiance unit. This was a change of 13.1% and 13.0% for *N*_0_ and *N*_1_*,* respectively. We should emphasize this difference in calibration coefficients is not likely due to instrumental drift as QWIP TIR arrays are stable over long periods of time [20]. The apparent changes in calibration coefficients are likely attributed to sensitivities in the uncertainties of atmospheric opacity and air temperature along the line of sight. Refer to Table 5 for complete calibration coefficient and emissivity results. Fig. 6 illustrates the comparison of the radiance derived from the camera DN values using our estimated calibration coefficients to the estimated radiance derived from the ground surface temperature data, our estimated emissivity and our atmospheric correction. The largest discrepancies occur during the hottest, followed by the coldest, times of the day.

**Table 5:** Calibration coefficients as defined in Eq. 6. ID is the sensor ID number. Change in the estimated camera calibration coefficient between the two days was ∼11%. RU stands for radiance unit in MKS.

ID	N_0_ (DN)	N_1_ (DN/RU)	e (All)	e (ROI Only)
All	-9304.05	1838.57		
B Cave				
9695783	-8727.04	1864.02	0.729765	0.677238
9695779			0.719626	0.656209
9695787			0.724459	0.668035
9695785			0.702367	0.644497
9695788			0.693667	0.635248
9695782			0.734446	0.675343
9695786			0.721647	0.648719
Station 7 trench				
9695781	-10521.1	2077.23	0.773473	0.739183
2233224			0.760042	0.730661
2233225			0.728786	0.700086
2041160			0.746743	0.715206

### Characterization of uncertainties and possible systematic errors

**Fig. 7:**
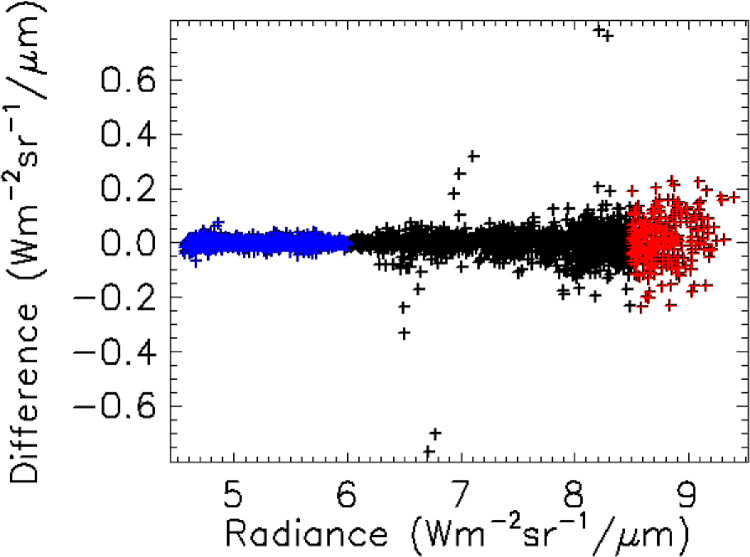
Estimated noise of the camera determined from comparing pixel DN to interpolated pixel DN. The blue region was used to determine camera noise when the estimated observed radiance was less than 6 radiance Wm^−2^sr^−1^µm^−1^ and the region used to determine camera noise when the estimated observed radiance was greater than 8 Wm^−2^sr^−1^µm^−1^. The results are shown in Table 6.

The QWIP camera's SNR and NEDT can be characterized using the data collected for this study. An upper limit for the noise can be determined by comparing the DN value to the linearly interpolated DN value based on the previous and subsequent DN values. The difference from these two DN values over a range of DN values was used to determine SNR and the NEDT, assuming the previously calculated calibration coefficients. Fig. 7 shows the distribution of “noise” as a function of DN. Radiance and equivalent temperature are also shown on the axis. Table 6 shows the results with an NEDT of ∼0.08° C and ∼0.48° C for brightness temperatures near 0° C and 20° C, respectively.

**Table 6:** Estimated camera noise based on DN levels at the 11 characterized sites.

Radiance Range (Wm^−2^sr^−1^µm^−1^)	Average Signal (Wm^−2^sr^−1^µm^−1^)	Standard Deviation (Wm^−2^sr^−1^µm^−1^)	SNR	Brightness Temperature for average signal (°C)	NEDT (°C)
<6	5.155	0.00961654	536.037	-3.85754	0.0815735
>8	8.76	0.0812977	107.784	21.5288	0.483917

The largest uncertainty in the calibration process described here is the atmospheric correction. The sampling of the air temperature at only one meter above ground level (AGL) and the absence of commercially available sunshades could allow for contamination of the air temperature measurements from radiance from the surface. This effect would be most prevalent during the mid-day, resulting in over-correcting for atmosphere effects. The potential for over-correction would reduce the estimated amount of radiance observed by the QWIP camera. The minimization approach used to determine both calibration coefficients and surface emissivity estimates could be affected. A systematic lowering of surface emissivity is possible. Additionally, any variation in atmospheric properties within the QWIP's FOV would be mapped into the emissivity parameters as there are site specific and the calibration parameters apply across the scene.

**Fig. 8:**
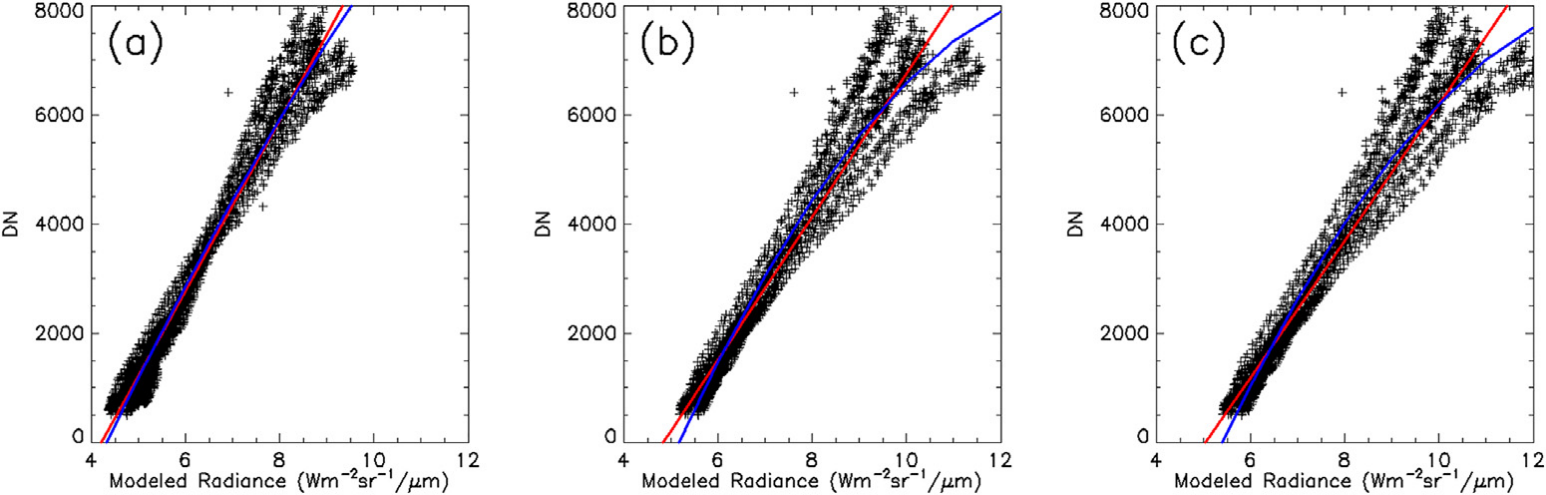
Image DN vs modeled radiance. These plots show a comparison of the image DN values for our 11 in situ sites compared to the modeled radiance using (a) the derived surface emissivity, a fixed emissivity of 0.85, and a fixed emissivity of 0.9. The red line shows a linear best-fit function while the blue line shows a quadratic best-fit function. For case that used our best-fit emissivity (panel a), the quadratic fit is nearly identical to the linear fit.

The next largest uncertainty may be the assumption that the surface temperature estimate is representative of the observed radiance across the entire pixel throughout the diurnal cycle. While we intentionally selected relatively large flat areas that were of uniform composition and texture, there were still local variations, such as rocks, that may be completely representative of the thermal response of the surface as measured by our in situ probes. The effect would potentially be mapped into both the thermal diffusivity estimate (through the effects on the estimated time lag between image pixel response to changing surface radiance and the in situ temperature measured) and the emissivity as the predicted radiance would slightly differ from the observed radiance. The issue of surface heterogeneity and the effects on estimated thermal physical properties is a well-known problem and has been a topic of entire papers (e.g., [1,3,7]) Further discussion of the uncertainty in emissivity and diffusivity estimates are discussed below. Emissivity estimates between the best fit using the entire dataset and the best fit using only B Cave ROI increased from 7 to 10%. Emissivity estimates between the best fit using all the data and the best fit using only Station 7 trench ROI increased by only 4%. For both ROIs, the estimated emissivity remained below the laboratory measured value of 0.86 for oblique views of basalt [2]. The oblique views in study ranged between 7° and 10°, and therefore could have even lower emissivity than previously measured. In order to determine the possible effects from underestimating, we conducted linear regression analysis comparing the estimated radiance, assuming a fixed surface emissivity of 0.86 and 0.9, to the observed DN. Fig. 8 shows that that there is significantly larger scatter for the fixed surface emissivity, when compared to our best-fit emissivity. This is not surprising as emissivity is the only observed radiance model parameter specific to each of the 11 sites. Both uncertainty of atmospheric conditions (air temperature and opacity along the line-of-sight) and possible heterogeneity of the surface in the pixel FOV could affect the best -fit emissivity. Regardless, our modeled atmospherically corrected observed radiance with variable emissivity, is a linear function to the observed DN levels.

The greatest uncertainty in determining the thermal diffusivity, α, is the uncertainty in estimating the time delay between instantaneous surface temperature (Image DN) and the measured near-surface temperature. These include intrinsic sensor lag and possible poor conductivity between probe and the surrounding subsurface matrix. These two uncertainties have the greatest effect for smaller time lags which corresponds to larger values of α. Because these uncertainties increase the estimated time lag, the estimated α from this source of uncertainty is likely a lower limit. As previously mentioned, non-uniformity of the surface within the pixel's FOV may not be completely representative of the location where the surface temperature is estimated. For example, any rocks present could either be warmer or cooler than the flat surface, depending on the time of day and whether the camera-facing rock face is shadowed or in direct illumination. Because of these effects, we recommend that in situ sensor sites be as uniform, flat, and rock free as possible. Ground truth of the surface thermal inertia would also have been useful for comparison so if practical, we recommend that samples be returned to laboratory facilities to measure *k, ρ*, and *c*. Estimates of these uncertainties are listed in Table S1.

*Thermal physical properties including implications for porosity:* Thermal inertia, especially for planetary surfaces such as Mars, is understood to reflect changes in grain size, where high thermal inertia correlates to larger grain size. This view is meaningful when considering surfaces composted of dust, sand, or cobble. At even higher thermal inertia, where the surface is competent rock, thermal inertia can be viewed as changes in porosity (or inversely, solidity). Robertson [31] showed that thermal conductivity was proportional to the square of solidity, while density was linearly proportional to solidity. We can combine these relations with Eq.s 5 and 6 to illustrate the relationship between thermal inertia, thermal conductivity, and solidity, assuming that the grain density and heat capacity of the surface remain *constant.*

(13) $$k=\alpha \gamma \rho_{o}c$$

(14) $$\Gamma =\sqrt{\gamma k\rho_{o}c}$$

**Fig. 9:**
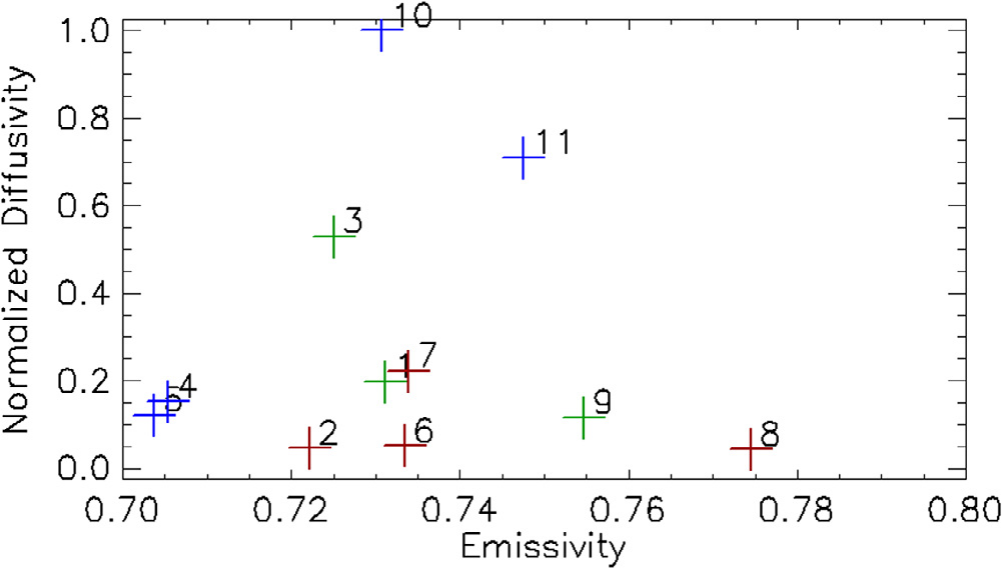
Normalized diffusivity vs apparent emissivity. The numbers located next to each data point correspond to the Supplemental Online Material (SOM) Fig.s, which show pictures of the landscape and the symbol color corresponds to composition – red: sandy mix, green: visible vegetation, blue: only basalt visible.

Where solidity is defined as γ = 1 - ϕ and ϕ is porosity. While beyond the scope of this paper, we suggest that diurnal thermal imaging, as presented here, could be used to determine near surface porosity in areas of uniform competent composition and slope, such as exposed outcrops of basalt or sandstone. Fig. 9 compares the previously discussed normalized thermal diffusivity to surface emissivity. If our scene had been relatively uniform in composition, the normalized thermal diffusivity would be a proxy for solidity. Further work is needed to validate this assertion.

### Findings and overflight planning

Using the methods elucidated above, we have shown (in Figs. 6,8) that there is a linear relationship between ground surface temperatures and the DN values of the QWIP thermal imagery, thus demonstrating that the thermal imagery acquired during the 2010 field campaign can be radiometrically calibrated to within a few percent within a diurnal cycle. We also found a day-to-day stability of better than 13%. As the dynamic range in DN values is ∼7000, we suggest that differences in pixel values between consecutive QWIP runs (such as day and night overflights), which are greater than ∼700 DN, are changes in the differences of thermal radiance and not due to shifts or drifts in calibration.

Furthermore, we believe the QWIP instrument used in this study had more stable calibration and linear response than our findings suggest. Better sampling of the line-of-sight atmosphere, as well as the use of commercially available radiation shields for atmospheric sensors were needed to improve the atmospheric characterization. Large oblique viewing angles also resulted in a range of *air mass*es between image foreground and background. This was observed for the B cave ROI. For the Station 7 trench ROI, viewing angles were less oblique and appeared to provide more consistent results.

Our results also demonstrated the QWIP's response was linear, and the day-to-day calibration was stable. While this could be easily demonstrated in a laboratory environment, those data may not always be available. Additionally, because of our findings, we suggest that QWIP images acquired of the same area, but on different days, can be directly compared without conversion of DN to surface temperature.

**Fig. 10:**
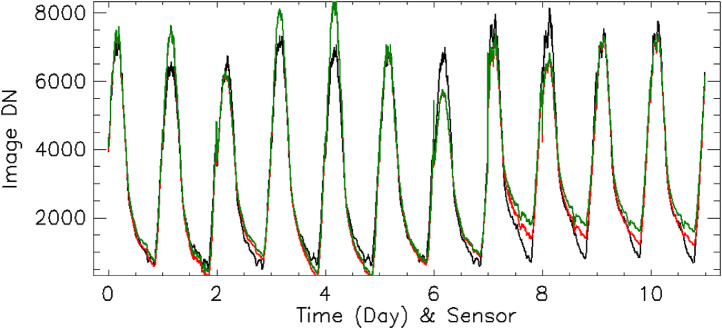
Estimated overflight DN levels. The black line is the DN extracted for our 11 sensor sites. Red line represents the model estimate of DN using our derived calibration coefficients and surface emissivity. The green line is the same model except all line-of-sight distances from the camera to the sensors have been adjusted to 800m, which simulate the expected results for an overflight. Pre-dawn DN levels are less than 2000 DN and mid-day DN levels usually exceed 6000 DN.

Finally, in Fig. 10, we used the derived calibration coefficients and estimated atmospheric correction to calculate the DN levels that would have been observed by an 800-meter overflight. There was a clear difference of more than 4000 DN that separated mid-day from pre-dawn observations. This compares favorably with the estimated DN variance for a 2-hour mid-day and 2-hour pre-dawn flight (Table 7).

**Table 7:** Pixel-to-pixel DN standard deviation for a 3 × 3 neighborhood.

Time of Day	S1	S2	S3	S4	S5	S6	S7	S8	S9	S10	S11
4:00-6:00	20.3	42.3	44.8	36.6	33.7	24.4	34.3	14.3	50.9	49.5	47.7
12:00-14:00	123.8	174.2	267	119.8	67.1	208.8	121.8	80	127.2	265.7	145.9

### Additional Information

As the cost of thermal cameras decrease and the quality continues to increase, the use of thermal imaging to characterize surface properties is likely to become more prevalent. While it is not always a viable option, the use of in situ temperature instrumentation provides a means of ground truthing camera calibration and enables researchers to characterize thermal physical properties of an area of interest. Previous studies using thermal cameras have often been limited to only a few times per day (e.g., [11,12]), and data were typically acquired during early afternoon and pre-dawn hours (to capture the diurnal maximum and minimum temperatures). These are also times when the surface temperature is relatively stable over a few hours flight window, which allows for overflight data to be collected and mosaiced. More recently, thermal imaging studies have expended temporal coverage. Herreid [14] used three-hour increments over multiple days to determine glacier melt under rock debris. High temporal resolution, such as this study, typically focus on short-term phenomena, such as cooling lava flows [33]. We hope that the study presented here will inspire additional high temporal resolution thermal imagery of diurnal or multi-day cycles for use in more general landscape characterization applications.

**Fig. 11:**
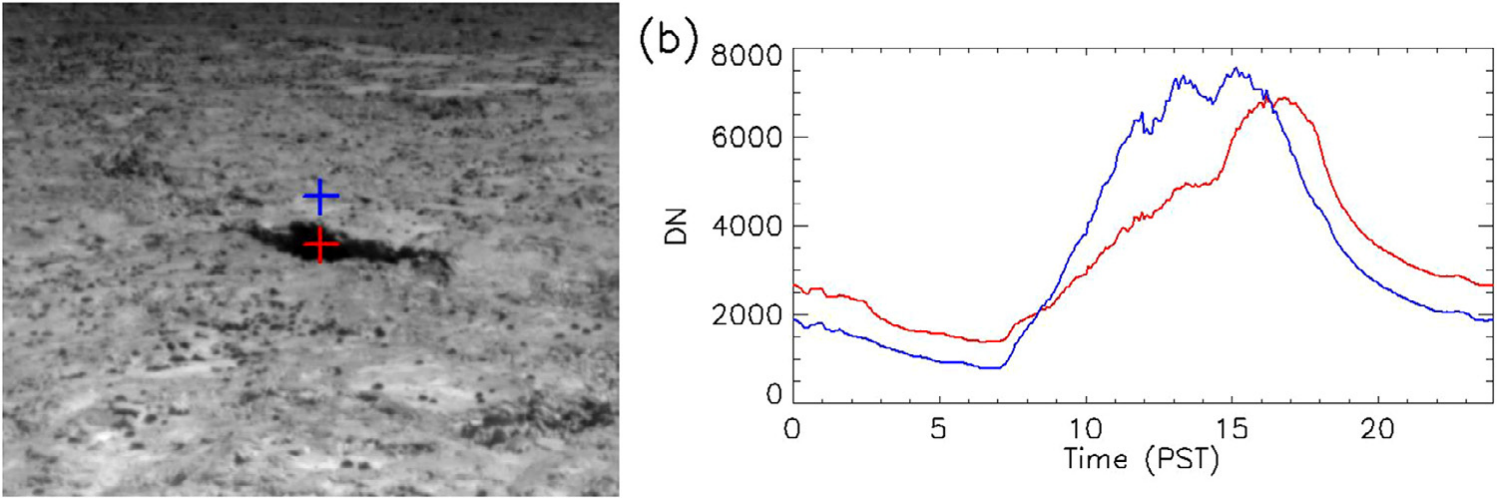
High temporal resolution thermal imaging shows slope effects typically missed in traditional mid-day and pre-dawn imaging. (a) Mid-morning QWIP image where the southwestern wall of the trench is in shadow. The plus signs indicate locations where temporal trends have been extracted and are shown in panel b. (b) The white line is from a fully sunlit surface, while the red line is in shadow. The trench has peak temperatures in the late afternoon. These important topographically induced shadowing effects could be overlooked when employing the traditional approach of acquiring data only twice per diurnal cycle.

While beyond the scope of thermal camera calibration, our results provide a window into thermal imaging of landscapes with significant topographic roughness – such as areas remaining in shadows that last throughout a significant portion of the sunlit hours. Fig. 11 illustrates this effect. To optimally acquire imagery in rough terrain, such as the Pisgah lava field, we recommend imagery be collected at least three times per a given diurnal cycle; this will enable researchers to account for and examine the effects of topographical shadowing. We further suggest mid-day, early evening, and predawn are optimal imagery acquisition times. Early evening has a lower cooling rate than late afternoon. Additionally, mid-morning data would have additional effects based on topography, but data collected from this time period would likely need to be corrected for rapidly increasing surface temperatures if images are acquired over a few hours and then used to construct mosaics. Another solution would be to conduct overflights with shorter flight windows, which is now possible with the advent of unmanned aerial systems. Perhaps the greatest potential for oblique high temporal resolution imagery is the use on other planetary surfaces. Rovers on Mars have already used thermal radiometers and spectrometers for remote sensing science [5,23]. Thermal imaging would be the next logical step.

## Declaration of Competing Interests

The authors declare that they have no known competing financial interests or personal relationships that could have appeared to influence the work reported in this paper.

## Appendix Supplementary materials

Image, application 1
