# Supplementary material for: Using near–surface temperature data to vicariously calibrate high-resolution thermal infrared imagery and estimate physical surface properties
Source: MethodsX. 2022 Apr 2;9:101644. doi: 10.1016/j.mex.2022.101644 (PMC9018161; doi:10.1016/j.mex.2022.101644)
Supplement: Supplementary file 1 [file mmc1.docx]

**Supplementary material *and/or* Additional information:**

Supplementary Table

Table S1: Summary of uncertainties and propagated errors.

| Physical Parameter, Symbol | Observable Measured, Symbol | Sources of error or uncertainty, Comments | Estimated Magnitude of error or uncertainty | Net Accumulated Error of measurement | Estimated propagated error to physical parameter |
| --- | --- | --- | --- | --- | --- |
| Thermal Diffusivity, α | Time Lag, Δt | Sampling Interval | ± 0.5 min | +0.5 min  -3.0 min | -0.15e-7  +0.95e-7 @1e-7 m^2^/s;  -1e-7  +10e-7 @19e-7 m^2^/s |
|  |  | Sensor response | - 0.5 min |  |  |
|  |  | Poor conduction between probe & regolith | - 3 min (likely an over estimate) |  |  |
| Surface Blackbody Radiance, b_s_ | Measured Temperature, T_meas_ | Sensor Accuracy | ±0.2 C | ±0.03@10°C  ±0.04@30°C  ~4% | <7% |
|  | Surface Temperature, T_srf_ | Thermal diffusion correction | ~10% of the diffusion correction | < 1% |  |
|  |  | Sensor measurement not representative of IFOV | ~10% of surface is 10C hotter | < 3% |  |
|  | Conversion of T_srf_ to Blackbody radiance | Spectral Response Function (SRF) | Deviation from a top hat to a delta func at 8.7 μm. | < 5% |  |
| Atmospheric Radiance, b_a_ | Atmospheric Temperature, T_atm_ | Sensor Accuracy | ±0.2 C | ~10% | ~10% |
|  |  | Sensor measurement not representative of entire line of sight. | ~10% |  |  |
| Atmospheric Transmissivity, τ | Relative Humidity, r | Sensor Accuracy | ±2.5% | ± 4% | ± 5% |
|  |  | Sensor measurement not representative of entire line of sight. | ± 3% |  |  |
|  | Distance, d | GPS Uncertainty | ±5 m | ± 2% |  |
| QWIP Modeled Radiance, R_obs_ (see Eqn 10) | Emissivity, e | Est. random error only (Fig S12) | < ±0.02 | <3% | ± 14% |
|  | Atm Transmit | Estimated Above | 5% | 5% |  |
|  | BB Srf Rad | Estimated Above | 7% | 7% |  |
|  | BB Atm Rad | Estimated Above | 10% | 10% |  |
| QWIP Observed Radiance, R_obs_ (See eqn 11) | Digital Number (DN), N | Background noise | ± 18 DN @ low DN  ± 150 DN @ high DN | SNR ~500 @ low DN  SNR ~100 @ High DN  See Table 6 | < 13%, as N0, N1 are not treated as independent variables. |
|  | Calibration Coefficient, N_0_ | See text | <13% | <13% |  |
|  | Calibration Coefficient, N_1_ | See text | <13% | <13% |  |
| Radiance Error,R_obs10_-R_obs11_ | Comparison of estimated radiance | See Fig 6. | 4% | 4% | 4% |

Supplementary Figures


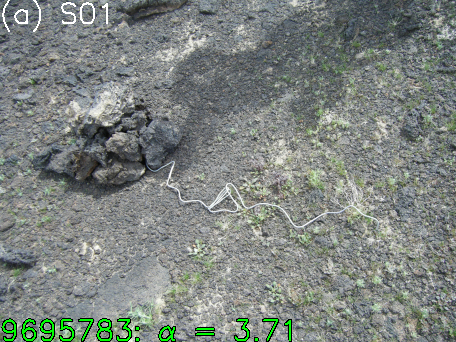


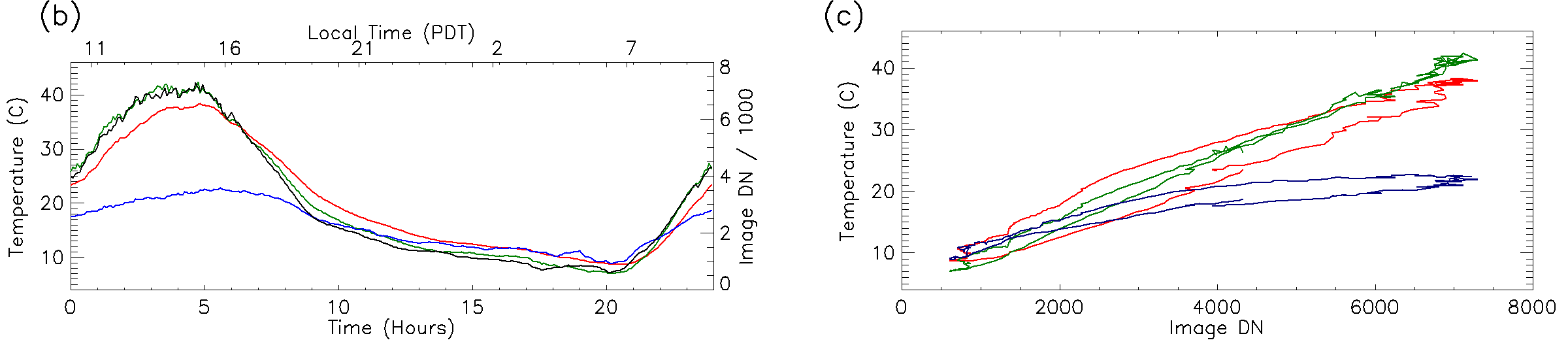


Figure S1: B Cave – 9695783. (a) Context image of the sensor location. (b) Temperature and image DN vs time, where time is in 5-minute increments. The black line is the image DN. The red line is the measured surface temperature. The green line is the corrected surface temperature. The blue line is the atmospheric temperature measured at ~1m above the surface. (c) Temperature vs. Image DN. The black line is for the measured temperature, the green line is using the corrected surface temperature, and the blue line is atmospheric temperature for reference.


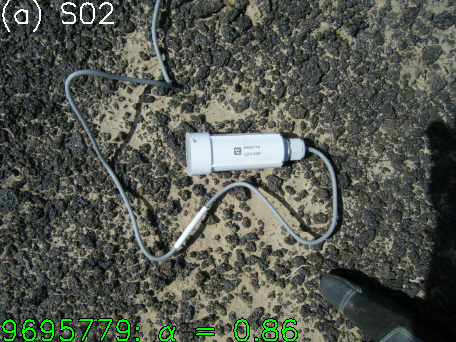


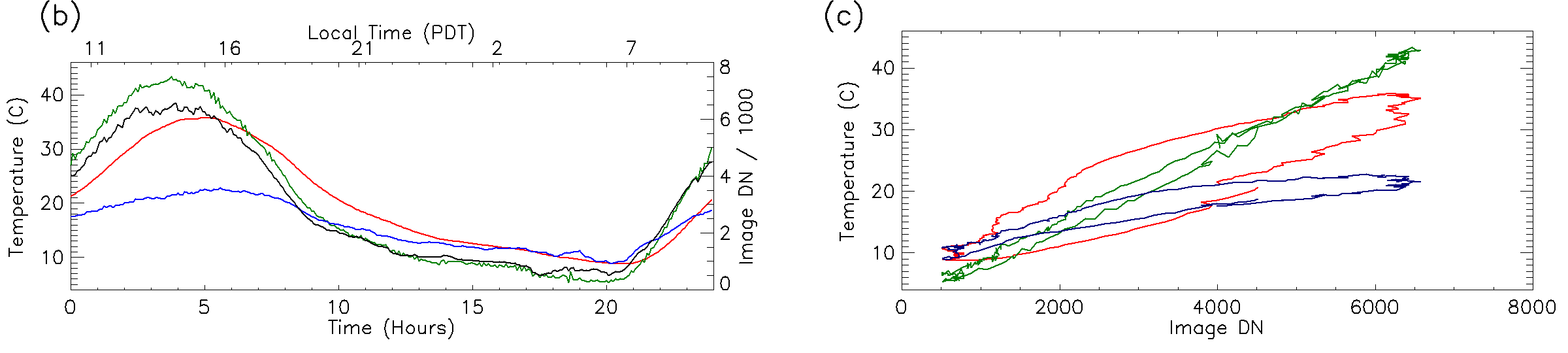


Figure S2: B Cave – 9695779. (a) Context image of the sensor location. (b) Temperature and image DN vs time, where time is in 5-minute increments. The black line is the image DN. The red line is the measured surface temperature. The green line is the corrected surface temperature. The blue line is the atmospheric temperature measured at ~1m above the surface. (c) Temperature vs. Image DN. The black line is for the measured temperature, the green line is using the corrected surface temperature, and the blue line is atmospheric temperature for reference.


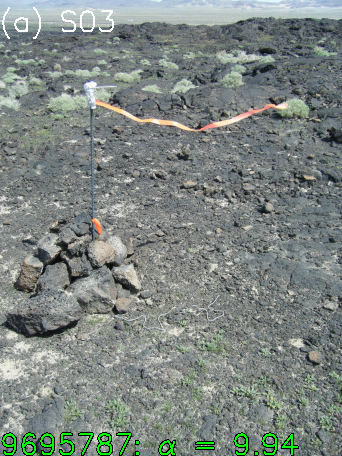


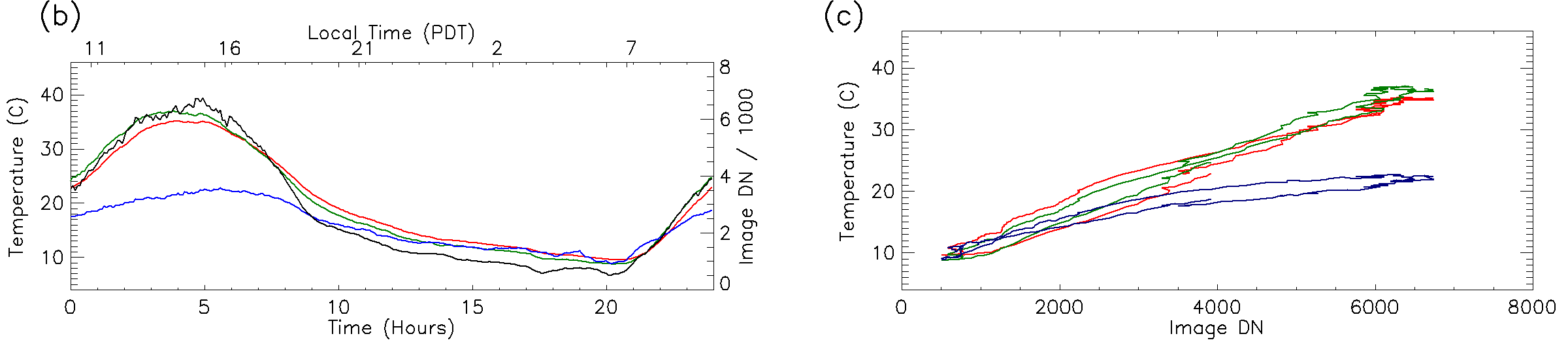


Figure S3: B Cave -- 9695787. (a) Context image of the sensor location. (b) Temperature and image DN vs time, where time is in 5-minute increments. The black line is the image DN. The red line is the measured surface temperature. The green line is the corrected surface temperature. The blue line is the atmospheric temperature measured at ~1m above the surface. (c) Temperature vs. Image DN. The black line is for the measured temperature, the green line is using the corrected surface temperature, and the blue line is atmospheric temperature for reference.


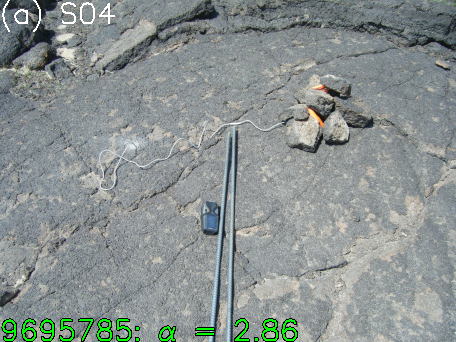


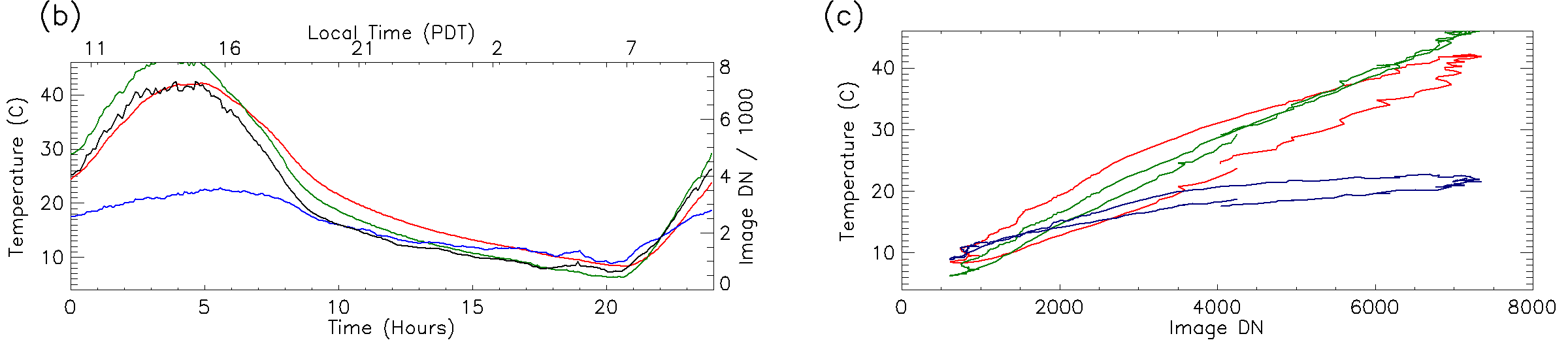


Figure S4: B Cave- 9695785. (a) Context image of the sensor location. (b) Temperature and image DN vs time, where time is in 5-minute increments. The black line is the image DN. The red line is the measured surface temperature. The green line is the corrected surface temperature. The blue line is the atmospheric temperature measured at ~1m above the surface. (c) Temperature vs. Image DN. The black line is for the measured temperature, the green line is using the corrected surface temperature, and the blue line is atmospheric temperature for reference.


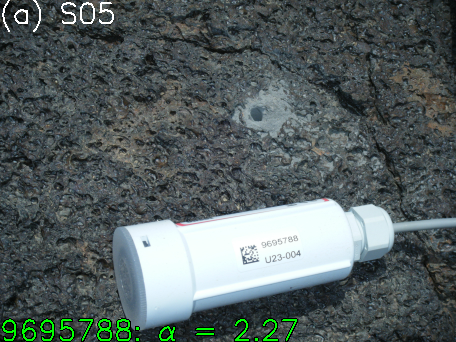


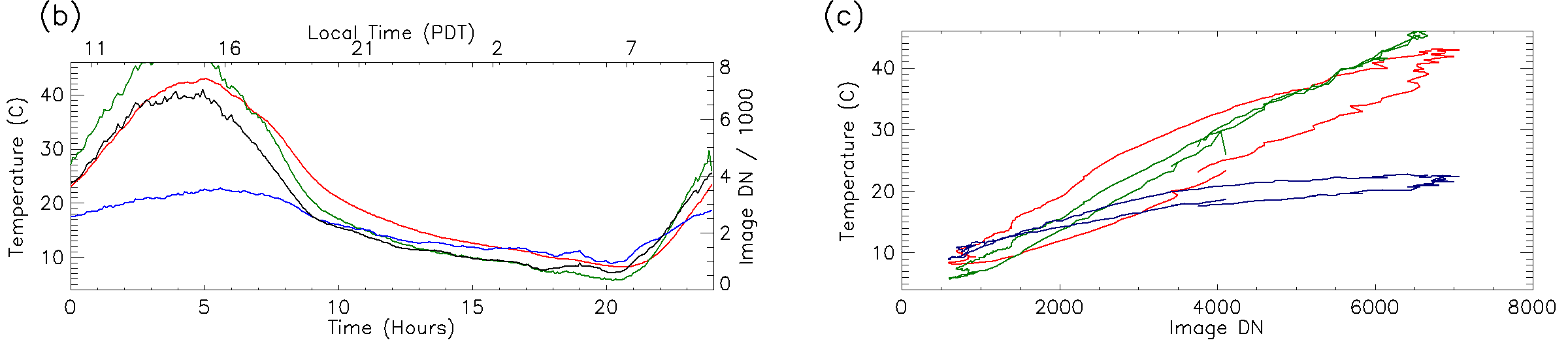


Figure S5: B Cave – 9695788. (a) Context image of the sensor location. (b) Temperature and image DN vs time, where time is in 5-minute increments. The black line is the image DN. The red line is the measured surface temperature. The green line is the corrected surface temperature. The blue line is the atmospheric temperature measured at ~1m above the surface. (c) Temperature vs. Image DN. The black line is for the measured temperature, the green line is using the corrected surface temperature, and the blue line is atmospheric temperature for reference.


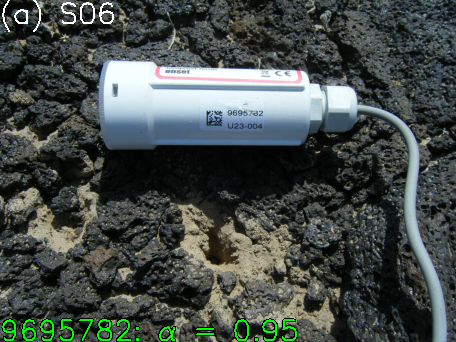


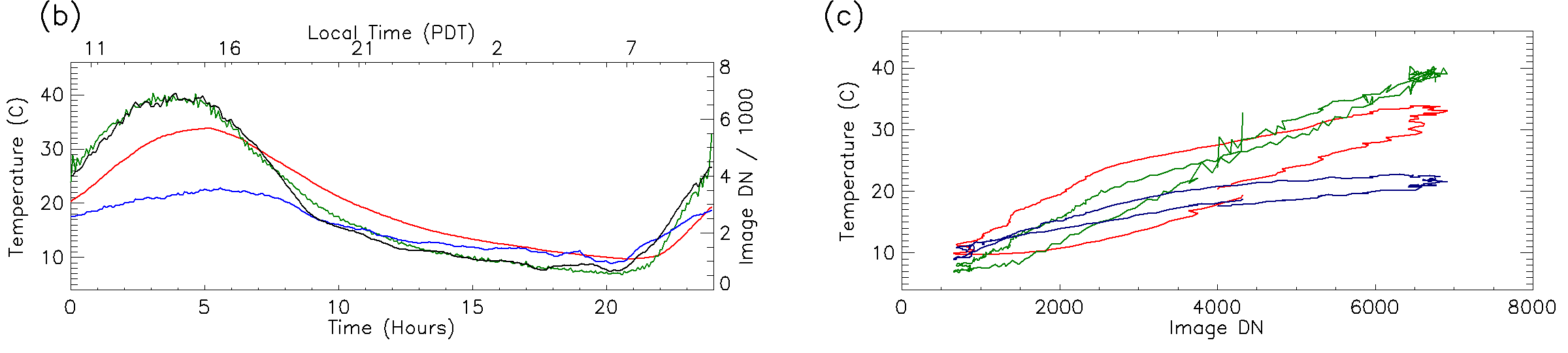


Figure S6: B Cave – 9695782. (a) Context image of the sensor location. (b) Temperature and image DN vs time, where time is in 5-minute increments. The black line is the image DN. The red line is the measured surface temperature. The green line is the corrected surface temperature. The blue line is the atmospheric temperature measured at ~1m above the surface. (c) Temperature vs. Image DN. The black line is for the measured temperature, the green line is using the corrected surface temperature, and the blue line is atmospheric temperature for reference.


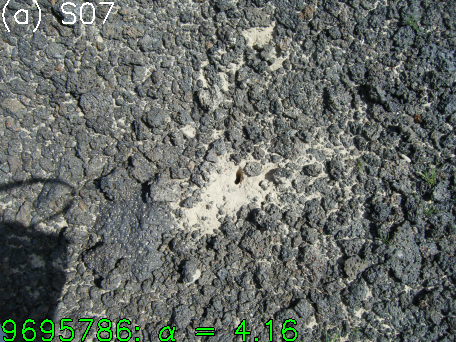


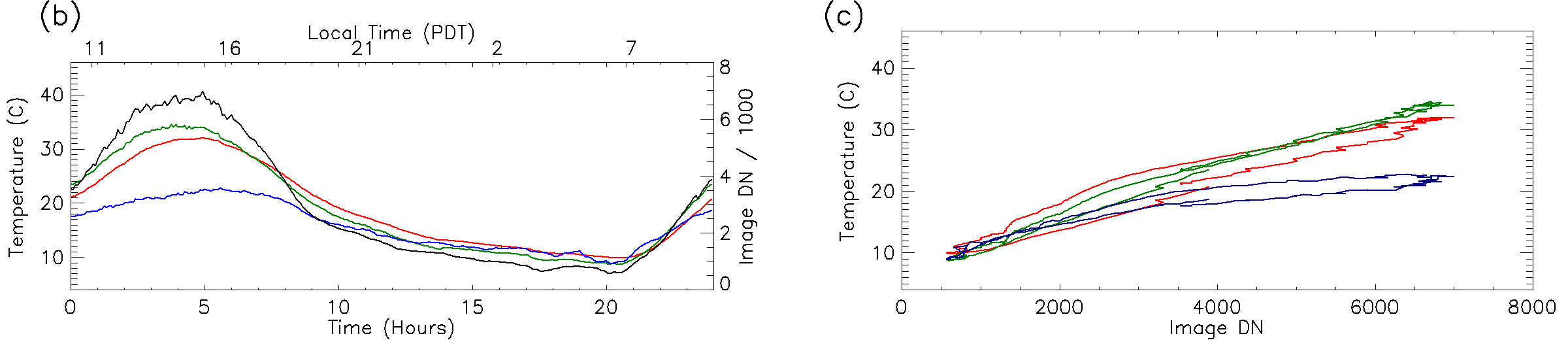


Figure S7: B Cave - 9695786. (a) Context image of the sensor location. (b) Temperature and image DN vs time, where time is in 5-minute increments. The black line is the image DN. The red line is the measured surface temperature. The green line is the corrected surface temperature. The blue line is the atmospheric temperature measured at ~1m above the surface. (c) Temperature vs. Image DN. The black line is for the measured temperature, the green line is using the corrected surface temperature, and the blue line is atmospheric temperature for reference.


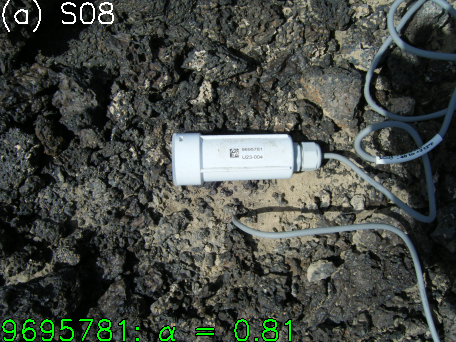


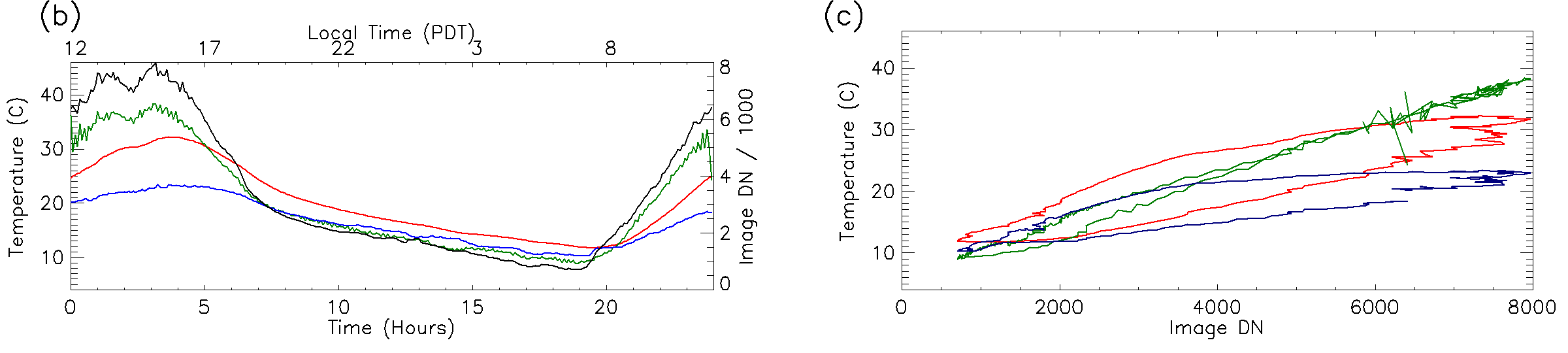


Figure S8: Station 7 trench - Sensor 9695781. (a) Context image of the sensor location. (b) Temperature and image DN vs time, where time is in 5-minute increments. The black line is the image DN. The red line is the measured surface temperature. The green line is the corrected surface temperature. The blue line is the atmospheric temperature measured at ~1m above the surface. (c) Temperature vs. Image DN. The black line is for the measured temperature, the green line is using the corrected surface temperature, and the blue line is atmospheric temperature for reference.


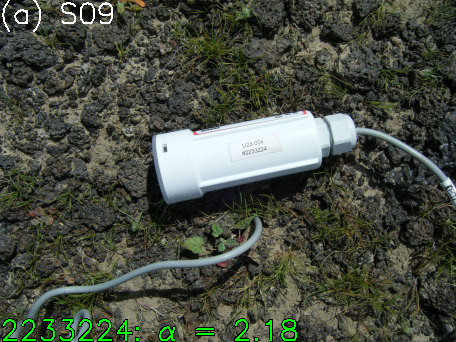


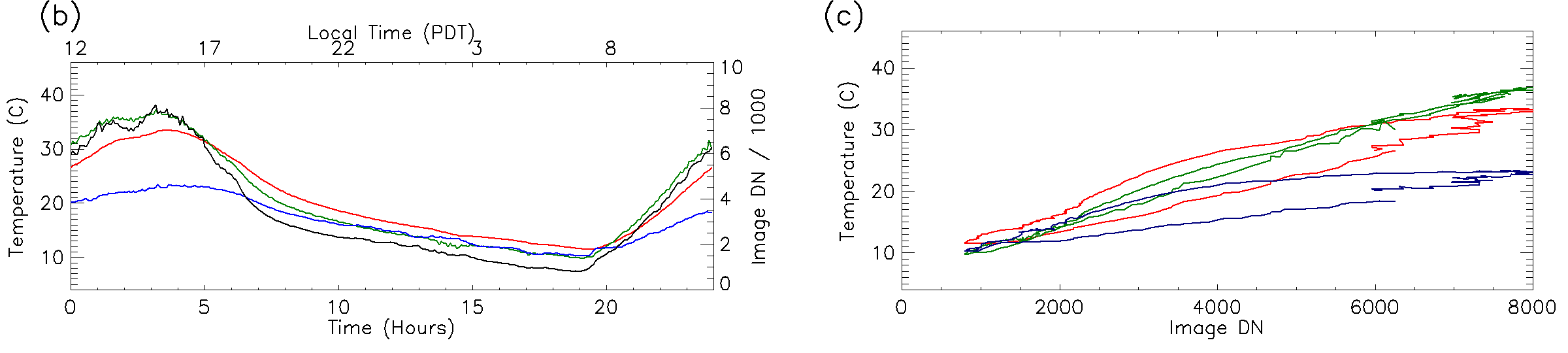


Figure S9: Station 7 trench - 2233224. (a) Context image of the sensor location. (b) Temperature and image DN vs time, where time is in 5-minute increments. The black line is the image DN. The red line is the measured surface temperature. The green line is the corrected surface temperature. The blue line is the atmospheric temperature measured at ~1m above the surface. (c) Temperature vs. Image DN. The black line is for the measured temperature, the green line is using the corrected surface temperature, and the blue line is atmospheric temperature for reference.


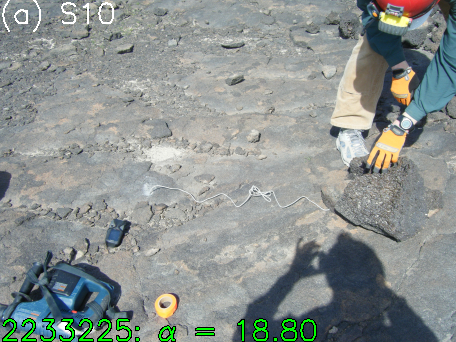


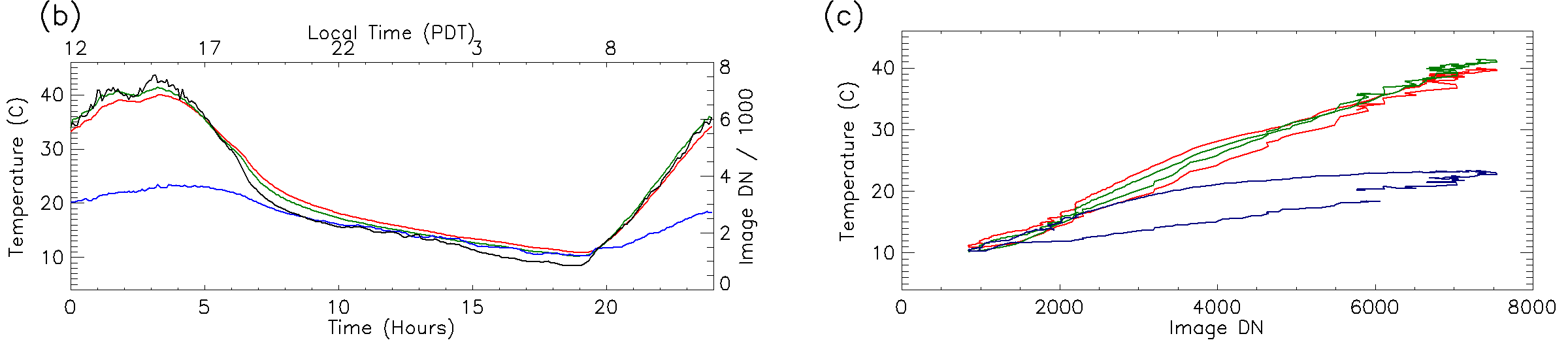


Figure S10: Station 7 trench – Sensor 2233225. (a) Context image of the sensor location. (b) Temperature and image DN vs time, where time is in 5-minute increments. The black line is the image DN. The red line is the measured surface temperature. The green line is the corrected surface temperature. The blue line is the atmospheric temperature measured at ~1m above the surface. (c) Temperature vs. Image DN. The black line is for the measured temperature, the green line is using the corrected surface temperature, and the blue line is atmospheric temperature for reference.


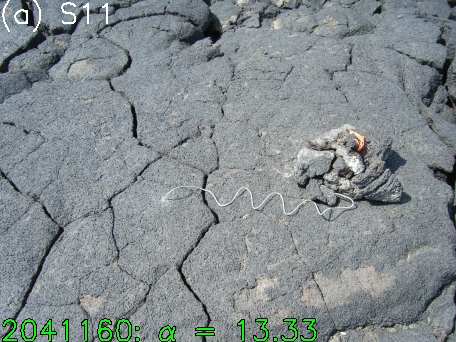


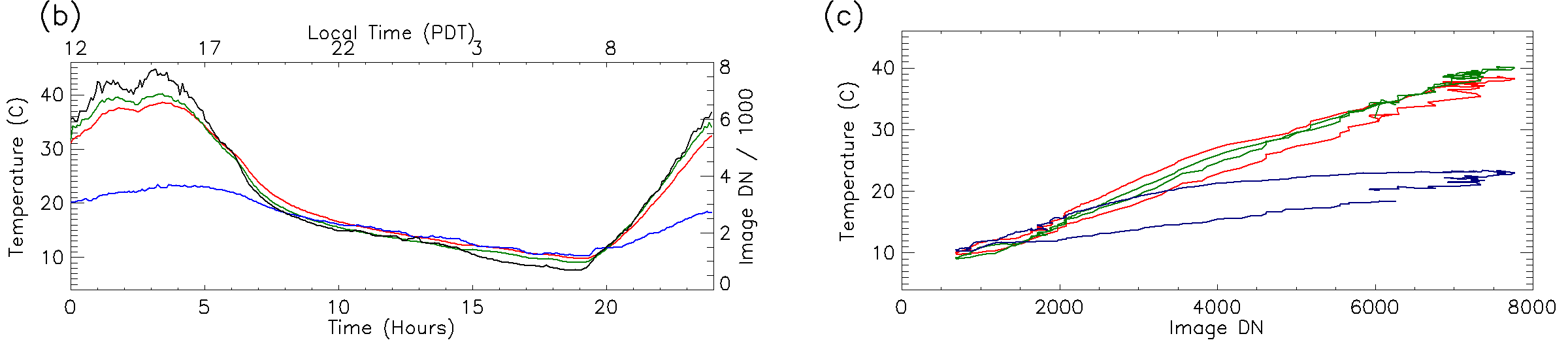


Figure S11: Station 7 trench – Sensor 2041160. (Upper panel) Context image of the sensor location. (lower left) Temperature and image DN vs time, where time is in 5-minute increments. The black line is the image DN. The red line is the measured surface temperature. The green line is the corrected surface temperature. The blue line is the atmospheric temperature measured at ~1m above the surface. (Lower right) Temperature vs Image DN. The black line is for the measured temperature, the green line is using the corrected surface temperature, and the blue line is atmospheric temperature for reference.


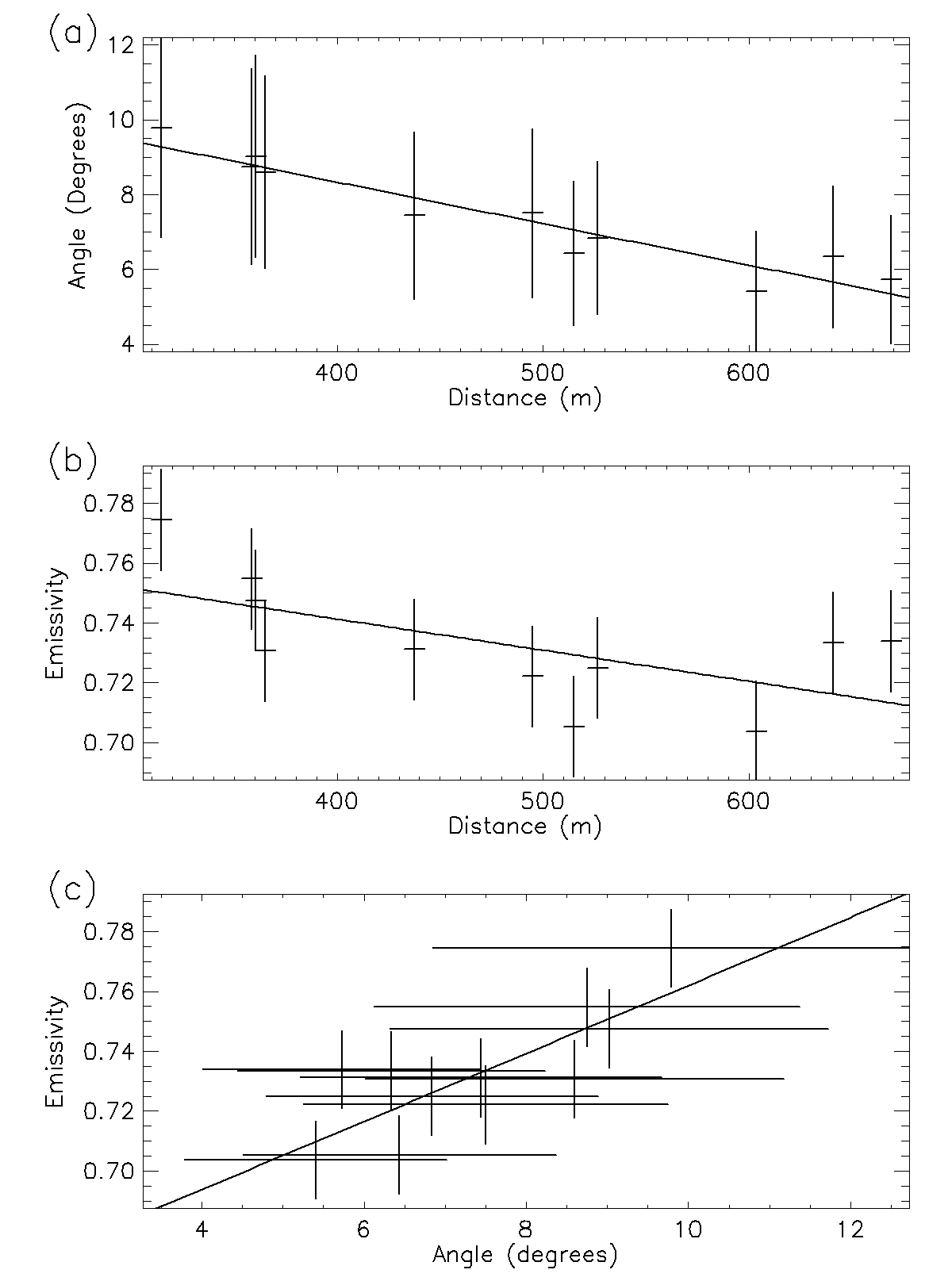


Figure S12: Comparison and estimated uncertainties between distance, viewing angle and emissivity. (a) Viewing angle vs. distance. The distance error bars assume 5 m uncertainty. The angle error bars assume a 30% uncertainty. (b) Emissivity vs. distance. The emissivity error bars are based on this best-fit linear regression with an estimated uncertainty of 0.017. (c) Emissivity vs. viewing angle. The emissivity error bars are based on this best-fit linear regression with an estimated uncertainty of 0.013.
